# Reviewing cancer’s biology: an eclectic approach

**DOI:** 10.1186/s43046-021-00088-y

**Published:** 2021-11-01

**Authors:** Ibrahim Diori Karidio, Senay Hamarat Sanlier

**Affiliations:** 1 Department of Biochemistry, Faculty of Science, E Block, Ege University, Erzene Mahallesi, Bornova, 35040 Izmir, Turkey; 2 ARGEFAR, Faculty of Medicine, Ege University, Bornova, 35040 Izmir, Turkey

**Keywords:** Lonal evolution model, Cancer stem cell, Cancer stem cell plasticity, Epithelial-mesenchymal transition (EMT), Cancer epigenetics, Microbiota, Mesenchymal-epithelial transition (MET)

## Abstract

**Background:**

Cancer refers to a group of some of the worldwide most diagnosed and deadliest pathophysiological conditions that conquered researchers’ attention for decades and yet begs for more questions for a full comprehension of its complex cellular and molecular pathology.

**Main body:**

The disease conditions are commonly characterized by unrestricted cell proliferation and dysfunctional replicative senescence pathways. In fact, the cell cycle operates under the rigorous control of complex signaling pathways involving cyclins and cyclin-dependent kinases assumed to be specific to each phase of the cycle. At each of these checkpoints, the cell is checked essentially for its DNA integrity. Genetic defects observed in these molecules (i.e., cyclins, cyclin-dependent kinases) are common features of cancer cells. Nevertheless, each cancer is different concerning its molecular and cellular etiology. These could range from the genetic defects mechanisms and/or the environmental conditions favoring epigenetically harbored homeostasis driving tumorigenesis alongside with the intratumoral heterogeneity with respect to the model that the tumor follows.

**Conclusions:**

This review is not meant to be an exhaustive interpretation of carcinogenesis but to summarize some basic features of the molecular etiology of cancer and the intratumoral heterogeneity models that eventually bolster anticancer drug resistance for a more efficient design of drug targeting the pitfalls of the models.

## Background

Although cancer is a common disease worldwide, its molecular pathology is characterized by a wide spectrum of biological aggressiveness that makes all the difficult of its control, thus a life-threatening disease. Etiologic studies’ reports about cancer support that its causal agent(s) could be heterogeneous ranging from genetic mutation (e.g., somatic mutation (s), inherited mutation (s), unavoidable DNA replication errors), to epimutation (hypomethylation of an oncogene (s), tumor suppressor gene silencing through hypermethylation, Chromatin remodeling), to viral infection (Hepatitis B/C, Human Papilloma Virus), exposure to aflatoxin B, *etc.* However, exogenous risk factors and/or lifestyle could also favor an early onset of certain types of cancers [1–4].

## Main text

### Carcinogenesis

Cancer occurs due to multistep and multifactorial molecular events that involve interactions between the genes and the environment of an organism through a process called carcinogenesis, also known as tumorigenesis or oncogenesis. The so-called process is a multistep process that involves sequential mutation(s) and/or epimutation(s), leading to uncontrolled cell proliferation and hedonic homeostatic dysregulation. Modifications causing cancer modulate stepwise cells metabolism and behavior. They alter their proliferation control, infinite lifespan, alter their communication with neighboring cells, and then confer them the ability to escape to the immune system. In brief, they are though damaged genetically and/or epigenetically, but they could divide and proliferate autonomously [5, 6].

Despite the differentiation process occurring within multicellular organisms, thereby generating a ubiquitous cellular diversity, cells function with common schemes in conducting fundamental processes that regulate cell proliferation and death. Cancer cells do not obey laws and rules that govern the intracellular corporation necessary for homeostasis in a multicellular organism. Consequently, cancer cells strive off to adapt, proliferate to invade nearby tissues, and fight off the defense system through their invasive aggressiveness. In 2000, Hanahan and Weinberg attempted to picturize the dense complexity of cellular alterations commonly found in cancer cells (six) as what they called *cancer hallmarks*. Since then, the concept has renewed impetus to cancer biologists and has been evolving. These include (i) permanently *switched on* the proliferative signal, (ii) evasion from growth suppressors, (iii) immortality/resistance to apoptosis, (iv) continuous replication, (v) angiogenesis induction, (vi) invasion and metastasis, (vii) dysregulation of cellular metabolism, (viii) overriding immune assault, (ix) genomic instability and mutations, and (x) tumor-promoting inflammation, immunoevasion [7–9].

Thus the fundamentals of cancer pathogenesis are cellular and molecular events. Indeed, defects in proper control of the cell cycle drive mechanisms responsible for a permanent *switch on* of carcinogenesis.

#### Cell cycle

The cell is defined as the anatomical and physiological unit of all forms of life. The cell is the unit of the biological community for multicellular organisms. Therefrom, cells fulfill all the characteristics of living things, namely functional organization, metabolism, homeostasis, growth and development, reproduction, passing on genetic information, responding to environmental changes, and adapting through evolution. Cell cycle refers to all the events that relate to cell birth, growth and development, and reproduction. It comprises the interphase (G1, S, and G2), stages, and cell division/mitotic (prophase, metaphase, anaphase, and telophase) stages. The ultimate aim of the cell cycle is strict accuracy in the DNA replication process and symmetric partition of genetic material in subsequent generation cells. The process involves molecular and structural events resulting in DNA replication (in S phase of the interphase), chromatin condensation, spindle formation for anaphase chromosome ascension, degeneration of the nuclear membrane, and reorganization of the cytoskeleton. Throughout the cell cycle’s process, there are regulatory checkpoints that control the conformity, compliance, and obedience of the molecular, and structural events occurring with respect to the ideal frame of the cell cycle. At the checkpoints, detected abnormalities trigger mechanisms leading to various alternative fates, namely a repair mechanism (DNA repair), cell arrest (senescence) pathways, necroptosis, and apoptosis. When a proliferating cell fails to activate either of these mechanisms in unicellular organisms, it leads to diminution of the reproductive capacity, while it leads to uncontrolled cell growth that potentially results in cancer in multicellular organisms. Two major types of control mechanisms govern the cell cycle, the first of which is extracellular and comprises the signaling pathways and intracellular mechanisms that are harbored by the checkpoints’ control [10–13].

##### Clinical significance of regulation and deregulation of cell cycle

In mammalian, the cell cycle is mainly governed by complex regulatory pathways to ensure correct cell division. Both extracellular and intracellular control mechanisms are involved in this regulation. First, for a cell to proceed to the G1 phase and pass the restriction-point (R-point), extracellular signaling, i.e., necessarily mitogens. After the extracellular signaling, the cell eventually passes through S, G2 phase, then through mitosis, but under intracellular controls. The latter control mechanisms are fundamentally and separately regulated by genetic programs, namely the checkpoints. These checkpoints assess the quality of DNA replication, its integrity, and the eventual progression of the cell through G2 and mitosis. A fundamental role in the cell cycle progression is orchestrated by regulatory and effector proteins and enzymes that eventually permit a carefully controlled series of biochemical reactions necessary for an ideal cell cycle. These regulatory proteins and catalytic enzymes could be cited as cyclins (A, B, D, E, and H), and cyclin-dependent-kinases (1, 2, 4, 6, or 7). The cyclins are a family of proteins that function as regulatory subunits for cyclin-dependent-kinases (CDKs) [14–16].

**Fig. 1:**
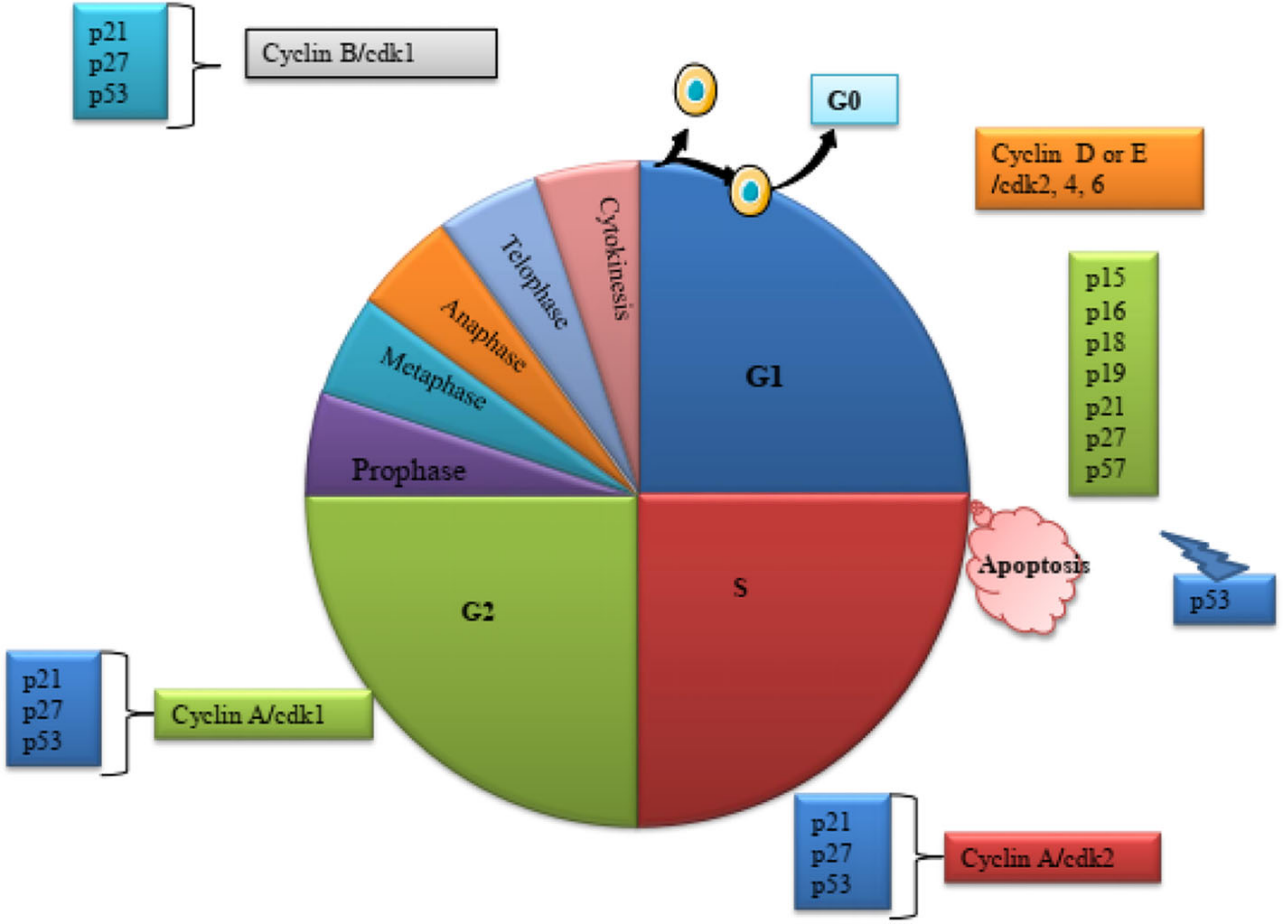
Dynamics of the cell cycle with its associated cyclin-cyclin dependent kinase complexes. The interphasic cells, at their G_1_ phase, are diploids (2*n*). As to prepare the cell for an eventual division, its genetic material, i.e., DNA, is replicated during the S phase. Thus in the later S phase and G_2_ phase, the cell has a doubled DNA (4*n*). The mitotic division occurs from Prophase through Telophase, as shown in the figure. At the end of mitosis, there is a formation of two daughter cells. G_0_ symbolizes a quiescent state through which cells could eventually go. The cyclin-cyclin-dependent kinases reflected on the figure are checkpoints of the cell cycle, each of which could eventually determine the cell’s fate with respect to the presence and/or absence of mitogens and growth factors

In contrast to yeast, vertebrates have up to four types of cyclin-dependent-kinases; the amount of each varies as the cell cycle proceeds from one phase to another. As such, there are G1-phase cyclins, G1-/S-phase cyclins, S-phase cyclins, and M-phase cyclins. Each of these cyclins forms a complex with cyclin-dependent-kinases to drive the cell through the subsequent phase (Fig. 1). While the cell synthesizes and/or degrades cyclins as the cell cycle proceeds from one phase to another, the amounts of the cyclin-dependent-kinases remain the same throughout the process. This is because as the concentration of a given cyclin falls, the corresponding cyclin-dependent-kinases become progressively inactive. Conversely, cyclin concentration increases are directly proportional to the activation of the cyclin-dependent-kinases of the corresponding phase [14, 17–20].

Some of the cyclin-dependent-kinases could bind to multiple cyclins, while for others, multiple cyclin-binding is not shared. For instance, CDK1 could bind to cyclin-A, B, D, and E, but has a higher affinity for cyclin B. CDK2 has a higher affinity for cyclin A and E, but could also bind to cyclin B and D. CDK4, and CDK6 have good affinity for cyclin D while CDk7 binds to cyclin H. Besides their regulation through the phase-specific expression and degradation of cyclins, cyclin-dependent kinases’ catalytic function is also controlled, as their name implies, by phosphorylation/dephosphorylation and by association with other proteins. The latter proteins are called cyclin-dependent-kinase-inhibitors (CDK inhibitors or CKI). They could either be of the family of INK4, i.e., specific to CDK4 (p15, p16, p18, p19) or CIP (p21) and KIP (p27, p57) that are efficient inhibitors to all CDKs [21–26].

In G1 phase, CDK-cyclin complexes drive the progression across the restriction point (R-point), through the phosphorylation of the retinoblastoma protein family members, namely Rb, p107, and p130. Early-mid G, histone deacetylase (HDAC) forms complexes with Rb and E2F. Phosphorylation of Rb by CDK-cyclins favors the release of E2F-DP transcription factors, and therefrom genes required for the cell cycle to proceed are expressed [27–30].

The components of the regulatory machinery of the cell cycle work in concert to ensure ideal conditions for an appropriate progression of the cell cycle. Besides their canonical regulatory role in the cell cycle, some of the members of the regulatory checkpoints have additional functions that would be located in the different cellular compartment(s). The most documented of the cell cycle’s regulators with additional function besides being a checkpoint of the cell cycle are the cyclins, cyclin-dependent-kinases, CIP, CKI, and E2F family proteins. These regulators could work either in concert as complexes as is the case in the cell cycle regulation or independently from one another. Cases that would exemplify these non-ministerial regulatory functions include (i) cyclin E1 works in concert with CDK2 to activate the transcription factor MYC thereby represses cellular senescence; (ii) CDK2, CDK4, acting in concert with cyclin D1, cyclin E1, cyclin A2, activate the transcription factor FOXM1C that in turn acts either, to promote the cell cycle promotion, or represses cellular senescence; (iii) cyclin D1, with its functional partner CDK4, represses NFR1 transcription factor, thereby inhibits mitochondrial functions; (iv) cyclin D1, alone acts as a repressor to the transcription factor FOXO1, O3 thereby inhibits anoikis; (v) the transcription factor JUN is activated by CDK6 acting alone as stimulation of angiogenesis; and (vi) cyclin D2, (D1, D3) represses the transcription factor DMP1, thereby modulate the p53 pathway, E2F regulates oxidative metabolism. To sum up, components of the regulatory machinery have non-canonical functions documented in various cellular processes, including cell differentiation, cellular senescence, cell death, cellular immune response, cellular metabolisms, mitochondrial activity, mitochondrial biogenesis, mitochondrial fission, and mitochondrial apoptosis. Beneath the cell cycle’s complex processes, there is an important requirement of energetic commitments provided by the mitochondria through an appropriate adaptive metabolism. Therefrom, the mitochondria and the regulatory machinery of the cell cycle entertain a functional dynamism that drives cell proliferation, survival, growth, or senescence [14, 31–36].

The cardinal characteristic of all neoplastic diseases is their pathognomonic feature that emerges from a dysregulated cell cycle that results in uncoordinated cell proliferation. Functional mutations and/or expressional dysregulation of the cell cycle regulators’ genes have been documented and proposed as oncogenic events. Therefrom, it has been of utmost importance to focus more interest on and have an in-depth understanding of the molecular control mechanisms of the cell cycle by its regulators. Abnormal functional activity and/or dysregulated gene expression of these regulators have been characterized in various cancers. The loss of functionality of some of these regulatory proteins does not hinder the subsequent cell proliferation in normal cells. However, the overexpression of others has been observed in many malignant neoplasms and is even used as a biomarker for diagnostic and prognostic purposes. Therefrom, genes governing the cell cycle control are categorized as oncogenes and tumor suppressor genes. Moreover, since aberrant gene expression patterns and/or functional activities of the regulators characterize cancer cells, scientists aim to take advantage of these specificities in designing selective therapeutics that would target them [34, 37–39].

#### Cellular models of cancer etiology

Mammalian organisms comprise mosaic cell populations in terms of their functions (e.g., differentiated cells), morphology, chemistry, and mitotic competence. Herein, the mitotic competence concerns us the best. Based on these criteria, three (3) categories of cells could be distinguished: actively cycling cells, cells that divide in response to stimuli (resting cells, stem cells), and cells would never divide again. Stem cells are those cells that divide for both the renewal of its type, and for producing cells that subsequently follow differentiation processes to eventually perform a given function (an asymmetric mitotic division of stem cells). Obviously, the clinical relevance of eventual aberrant functionality and/or dysregulated gene expression of the cell cycle regulators in the respective pre-cited cell types is not the same. In fact, only a few cell types have the ability to proliferate and give rise to a tumor eventually. Per analogy to the mosaicity of normal organismal cells, tumors are also characterized by intratumoral heterogeneity. That is to say that cells of a tumor are heterogeneous in their nature with respect to their morphology, chemistry, and malignant potentialities. In fact, there are two popularly established models, and a third establishing model of cancer cells mosaicity, namely the clonal evolution model, the cancer stem cell model, and the phenotypic plasticity/plastic cancer stem cells model, respectively. These models have supported scientific reports that shed light on their similarities, and differences with their respective beneficial clinical implications. The growing knowledge brought about by the investigation around these models gives rise to a better understanding of cancer, and allows for the development of better efficient therapeutics, and preventive protocols [40, 41].

##### Cancer stem cell hypothesis

**Fig. 2:**
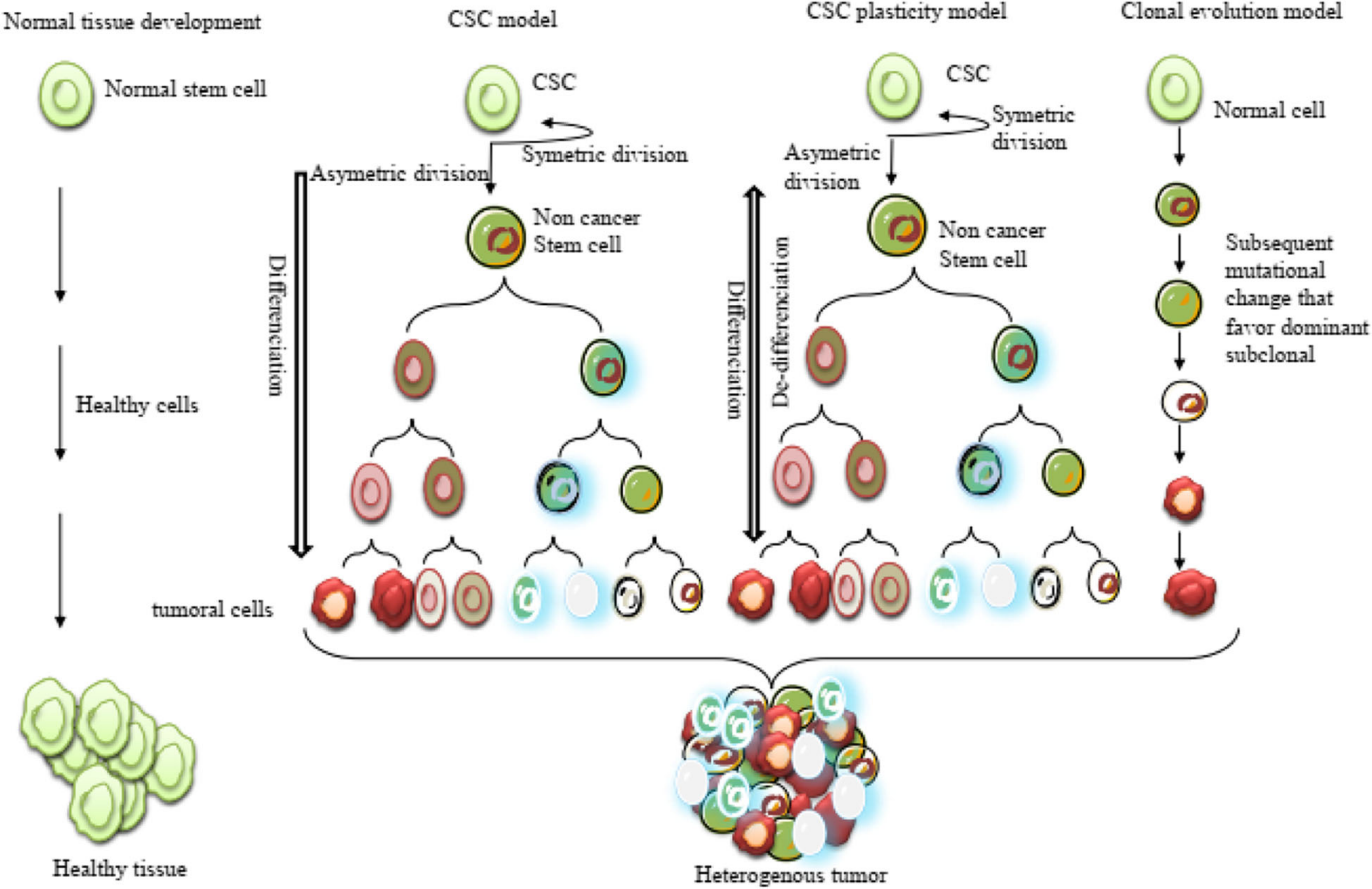
Intratumoral heterogeneity models. The clonal evolution model suggests that intratumoral heterogeneity emerges from Subsequent mutational change that favors dominant sub clonal cells. Intratumoral heterogeneity sets up through cell differentiation according to the cancer stem cell model. The cancer stem cell plasticity model supports that intratumoral heterogeneity could be sourced from dedifferentiation of neoplastic cells

According to the cancer stem cell hypothesis, there is a subset of cancer cells with the characteristics of stem cells. They drive cancer initiation, progression, metastasis, and eventual recurrence following treatment. The cancer stem cells are featured with the particular ability to divide both symmetrically for self-renewal to ensure perennity of undifferentiated cancer stem cells, and asymmetrically to generate progenies that subsequently, and irreversibly differentiate thereby, establishing the hierarchical organization of the tumor as per analogy to normal tissue. Also, as supportive arguments to this model, the cancer initiation cells are derived from normal stem cells, or progenitor cells that would have acquired cancerous changes (mutation/epimutation). According to this model, cancer is a stem cell disorder. The significant particularities of malignant stem cells are that they are vested with dysregulated stemness pathways, while healthy stem cells obey homeostatic stemness pathways with stable genetic and epigenetic profiles (see Fig. 2). These cells are also endowed with quiescent properties that would explain cancer metastasis, and the late relapses that characterize cancer’s resistance to radio- and/or chemotherapy. Traditional cancer therapy, i.e. radiotherapy, and chemotherapy, broadly target pathogenically proliferating cells without any discrimination. Cancer stem cells, and non-tumorigenic cells have different sensitivity to therapy. Cancer stem cells have a great ability to resist therapeutic assault. This might be partially due to their high content of free radical scavengers enabling them to remediate DNA-damages following DNA-targeting therapy eventually. Therefrom, the isolation, and the study of the mechanistic pathways through which therapeutic resistance develops in cancer stem cells would likely help us to understand how tumor recurrence occurs, and eventually drive the design of targeted therapy directed to these pathways specific to malignant stem cells. However, there is a growing body of evidence that shapes the non-universally applicability of the cancer stem cell model to the complex heterogeneity of malignancies with respect to therapeutic challenges, thus raises issues, of clonal cancer evolution, of EMT and the clinical significance of cancer microenvironment [42–48].

##### Tumor microenvironment

Tumors could be distinguished as solid tumors, i.e., localized (at least in their early stages) as a proliferating mass of cells in a given tissue and tumors like leukemia and lymphomas that occur in lymphatic tissues, namely blood and lymph, respectively. Structurally, per analogy to healthy tissues, solid tumors comprise the malignant cells that constitute the parenchyma and the stroma (microenvironment) within which the neoplastic cells are enveloped by the basement membrane surrounded by the tumor microenvironment. For leukemia and lymphomas, the blood and lymph, respectively, constitute their stroma. Scientific evidence supports that these structures are interdependent at all stages of cancer and develop in parallels. Tumor stroma consists of stromal cells (genetically stable cells), the basement membrane, and the non-cellular matrix also called the extracellular matrix (ECM). Carcinogenesis occurs through multistage patterns; the first is an environmental assault (initiation), followed by promotion events. Cancer stem cells, localized in their niches (privileged anatomical sites), entertain a bidirectional communication with the neighboring microenvironment which is vital, and determine the progression/metastasis for primary tumors and dormancy/proliferative status for metastasized malignant stem cells. Therein, tumoral cells secrete various biomolecules (growth factors, cytokines, proteases...) to induce local microenvironmental modulations that result in perturbation of the physicochemical properties of the host tissue. It has been suggested that the correctional remodeling of the tumor microenvironment would have normalizing effects on tumoral cells, thus avoiding their ablation just through stromal cells re-education. Tumor stromal cells include adipocytes, cancer-associated fibroblasts (CAFs), myeloid-derived suppressor cells, natural killer cells, T-regulatory cells, perivascular cells, neuroendocrine cells, and others like mast cells. The extracellular matrix is a complex collection of macromolecules with various biochemical and biomechanical properties. Components of the extracellular matrix include proteins, proteoglycans, glycoproteins, and polysaccharides. The extracellular matrix is tightly controlled through embryonic and organ homeostasis but becomes commonly dysregulated in neoplastic diseases favoring anarchic cell proliferation and metastasis [49–53].

The process briefly described would start with the cancer stem cells secreting growth factors and cytokines released in the host tissue microenvironment. For instance, in breast cancer, the cancer stem cells induce the proliferation of cancer-associated fibroblasts using the release of secreted platelet-derived growth factors (PDGF). Subsequently, cancer stem cells activate cancer-associated fibroblasts that, in turn, synthesize collagen I and II, components of extracellular matrix and connective tissue, or stroma that characterize solid tumors. The so-formed tumor stroma is irrigated by blood vessels that provide tumor cells with oxygen, nutrients, and evacuate waste. For the ongoing course of remodeling the stroma, cancer cells activate cancer-associated fibroblasts through the secretion and release of TGF-β. The activated cancer-associated fibroblasts then release metalloproteases (MMPs) to digest collagen fibers, which make up connective tissue. Cancer-associated fibroblasts would also eventually produce HGF that would drive motility and invasive behavior of tumor cells [54–56].

##### Clonal evolution hypothesis of cancer cells’ heterogeneity

The current evolutionary biology of carcinogenesis originated from Nowell’s “clonal evolution” model of cancer cells suggested in the 1970s. According to this model, cancer emerges from a single mutated cell, which, as it divides, undergoes subsequent additional alterations to generate subpopulations of cells, the biological and clinical fate of which is determined by the fitness acquired over time and natural selection. Thus the clonal evolution model could be pictured as a succession of clones of cancerous cells. Each new clone appears by acquiring new mutations conferring a selective advantage: the resulting clonal diversity sets the basis for the intratumoral heterogeneity [57–61].

The conceptualized clonal evolution model of tumors explains how mutation (s) and/epimutation (s) of oncogenes/tumor suppressor genes would drive carcinogenesis. However, neither the cancer stem cell model nor the clonal evolution model could universally apply to all cancers. The common caveat to the “clonal evolution” model lies in the hierarchical organization of tumor cells, the *Sine qua non* of the “cancer stem cell” model. Therefrom, when the intratumoral heterogeneity emerges from mutational and epimutational events, clonal evolution would be the most appropriate model to address this tumor. However, some cancers would require both models to explain their inhomogeneity, while others would require either/none of them. The initial stages of carcinogenesis could start by following a cancer stem cancer model, then subsequently moves on with the clonal evolution model. In fact, if an environmental insult induces selective phenotypical and/or functional mutation (s)/epimutation (s) into a growing tumor, depending on the stage at which it occurs, the fate of the intratumoral heterogeneity with respect to its model would change: (i) if only cancer stem cells are affected, then the eventual intratumoral heterogeneity sets right among cancer stem cells (cancer stem cell model); (ii) if the alterations affect non-tumorigenic cells, it would not be transmitted, provided the tumorigenic status has not been changed (cancer stem cell model); and (iii) if the alterations affect differentiating tumorigenic cells, then the cancer stem cell and clonal evolution models would be required to address the resulting intratumoral heterogeneity [58, 62–65].

##### Epithelial-mesenchymal transition, mesenchymal-epithelial transition, and cancer stem cell plasticity

**Epithelial-mesenchymal transition nd mesenchymal-epithelial transition** Epithelial to mesenchymal transition (EMT) is an integral physiological process of embryogenesis first described in the 1980s by the American Developmental Biologist Betty Hay. During this process, the immotile polarized epithelial cells, through biochemical and epigenetic events, progressively lose some of their inherent characteristics as they are gaining mesenchymal cells’ properties, i.e., as they are becoming motile invasive potentiated stromal cells. However, changing from epithelial to mesenchymal cells does not alter their inherent plasticity. Therefrom, both mesenchymal and epithelial cells are pluripotent cells. EMT is exemplified by various homeostatic physiological and developmental processes such as wound healing, tissue regeneration, myogenesis, gastrulation, palatogenesis, heart valve formation, etc. [35, 66–68].

The reverse process of EMT, i.e., mesenchymal to epithelial transition (MET), is also a physiologically observable process. MET refers to the phenotypic plasticity events that involve the dedifferentiation of motile, nonpolarized, and potentially invasive mesenchymal cells to nonmotile polarized epithelial cells joined to one another and packed onto the basement membrane. Both EMT and MET are rigorously controlled physiological processes observed but not properly controlled in disease conditions such as inflammation, fibrosis, and cancer metastasis. Consequently, EMTs are classified into three biological subtypes: type I EMT, type II EMT, and type III EMT. Type I EMT is observed in embryogenesis and organogenesis; type II EMT is seen in fibrosis, wound healing, and tissue regeneration; and type III EMT is observable in tumor metastasis [69–72].

**Molecular mechanisms of epithelial-mesenchymal transition in cancer** The molecular mechanisms governing EMTs have common and highly conserved hallmarks, although each of the three biological subtypes has specific mechanistic features. In general, cells undergoing EMTs are subjected to genetic, epigenetic, and biochemical events that result in phenotypic and gene expressional changes. In cancer, the process has been exclusively described in carcinomas, i.e., the most diagnosed forms of cancers. The process follows a multistage program that involves intracellular reorganization and cell-surrounding events that could be summarized as (i) detachment and loss of polarity of the epithelial cells recruited to undergo EMT; (ii) local digestion of the basement membrane, intravasation then extravasation through the lymphatic or blood circulatory system; and (iii) tissue invasion and dormancy [70, 73, 74].

During embryogenesis, EMT mediates morphogenesis and stem cell plasticity; in wound healing, it favors tissue regeneration; and in physiopathological conditions, as it is the case in cancers, it mediates the most ghastly phase of cancers, i.e., metastasis. Thus EMT is similar to a crossway of physiological, homeostatic, and pathophysiological pathways in human life that eventually respond to intrinsic (mutation, epimutation), and extrinsic (growth factors, immune response) stimuli. Prior to metastasis, tumoral cells have cohesive adhesion with one another and with the neighboring microenvironment until they start releasing the colony-stimulating factors (CSFs), to recruit macrophages as well as other stromal cells. The macrophages, in turn, respond through the release of epithelial growth factors (EGFs), thereby promoting a positive feedback loop with tumoral cells releasing more CSF. Macrophages subsequently release cathespin B that generates the primary hallmarks of EMT [75–78].

The primary hallmarks of EMT reflect the loss of EMT markers, namely E-cadherin, desmoplakin, Mucin-1, occludin, and cytokeratins (i.e., CK8; CK18; CK19). E-cadherin is a transmembrane protein coded by the *CDH1* gene that happens to be a tumor suppressor gene but also playing supplementary roles in cell polarization, cell differentiation, cell motility, and cell’s stemness properties. Extracellular portions of E-cadherin of neighboring cells interconnect while their intracellular tails bind to actin filaments by means of alpha-catenin. During cancer progression and metastasis, CDH1 undergo functional mutation or epimutation (i.e., hypermethylation of the gene’s promoter), or transcriptional amplification of the repression factor. Consequently, epithelial cells locally lose their cell-cell adhesion and basal-apical polarity. Therefore, they are allowed to detach and migrate toward the adjacent tissue as their neighboring cells undergo morphogenic rearrangement to fill the gap. Stromal cells release matrix metalloproteases (MMPs) to digest the basement membrane, thereby generating a chemical gradient that results in the cytoskeletal actin rearrangement by the chemoattractant, i.e., hepatocyte growth factors via *Ras*-like GTPases. The cytoskeletal actin fibers reorganize to stress fibers and subsequently form lamellipodia, filopodia, and invadopodia, each of which has an especially vital role to play during cancer metastasis. Lamellipodia are wheel-like actin filaments that mediate the cell’s mobility. Filopodia are spiky membrane protrusions that sense the surrounding of the cell. Invadopodia produce proteases containing vesicles to degrade the extracellular matrix [75, 79–82]

During the ongoing process, macrophages, alongside EGFs, produce tumor necrosis factor α (TNFα), to downregulate the synthesis of epithelial E-cadherin (through upregulation of its transcriptional repressors a.k.a. as EMT-TFs, namely SNAIL, SLUG, ZEB1, ZEB2/SIP1), and upregulate the synthesis of mesenchymal N-cadherin. Thereon, epithelial genes are progressively “switch down” as mesenchymal genes “switching on” to allow the phenotypic transition from epithelial to mesenchymal along with the characteristic features. They eventually acquire invasive abilities as well as resistance to senescence and apoptosis [83–86].

Therefore, those cells enter the circulatory system through intravasation. During their journey through the circulatory system, they are bound to platelets to protect them from immune attacks. They set the house to the metastatic site following extravasation from the circulatory system at the end of their journey. It is believed that prior to colonization of the secondary site, the metastatic cells undergo MET. On the metastatic site, two scenarios are possible: (i) either the metastatic cells successfully colonize the secondary site and grow as a secondary tumor after a latent phase called tumor dormancy that would last for years; (ii) or they revert to normal epithelial cells [87–89].

**Cancer stem cell plasticity** CSC plasticity model correlates tumor's hierarchical-morphological, phenotypical, and functional heterogeneity to EMT that mechanistically mediates dynamic and bidirectional (re)-population of the CSC pool that essentially characterizes tumorigenicity. Let us recall that the CSC model posits that tumor’s heterogeneity emerges from the hierarchical organization of tumor, which results from differentiation of a portion of CSCs. On the other hand, clonal evolution supports that cells acquire evolutionarily transmissible mutations through the sequential course of tumorigenesis; therefrom tumor heterogeneity emerges. CSCs’plasticity model, by means of EMT/MET, somehow combines the intrinsic features of both CSC and clonal evolution models. In that, it incorporates the fundamental hierarchical organization suggested by the CSC model, but with an eventual dynamic bidirectional interconversion of CSCs to non-CSCs. From this prospect, EMT is like the knob to three fearsome outcomes of carcinogenesis: bidirectional interconversion of CSC-to-non-CSC (setting up the hierarchical organization of tumor cells), non-CSC-to-CSC (regeneration of the CSC pool following chemotherapy), and metastasis. Also, EMT confers non-genetic plasticity that mediates metastasis of a tumor reported in the clonal evolution model. The portion of a clonal evolution model of a tumor that undergoes EMT would have stemness properties that eventually result from dedifferentiation. Diversely, the plasticity of CSC represents only a fractional aspect of cancer cells’ plasticity, i.e., the scope of tumor cells’ plasticity is far beyond that. It is believed that more aggressive CSC could emerge from epimutation(s) following an aggressive therapy. Moreover, a tumor with historical EMT would have CSC, non-CSC, EMT-CSC, and MET-CSC subpopulations [90–95].

#### Mitotic catastrophe

There is no definitive consensus on the definition of mitotic catastrophe. Historically, the “microtubule catastrophe” reported by Mclntosh in 1984 and the temperature-dependent lethal abnormal chromosome segregation reported in 1986 in *Schizosaccharomyces pombe* by Russell & Nurse settled basic criteria for defining mitotic catastrophe [96, 97].

Mitotic catastrophe is a set of events that characterize a cell death type induced by cell exposure to radiation, chemotherapeutic drugs, or hyperthermia. The rather complex mechanistic signaling that triggers mitotic catastrophe is not fully understood, but the process is believed to occur subsequent to aberrant (partial disintegration of anaphase nuclei), or premature mitosis. This is a mechanism of eliminating incompetent mitotic cell distinct from the programmed cell death-apoptosis in that mitotic catastrophe occurs in cells with functionally defective apoptotic pathways and deficient in p53. Cells that undergo lethal damage following exposure to radiation or cytotoxic drugs during their interphase may never enter mitosis. Subsequently, rapid cell death usually occurs through apoptosis. Otherwise, damaged cells entering mitosis terminate with aberrant mitosis at metaphase or anaphase. The aberrant mitosis does not properly segregate chromosomes and results in forming a non-viable cell with double or more nuclei that are partially or completely separated from one another [96, 98–100].

The great importance of the mitotic catastrophe lies in its clinical significance. Many well-documented anticancer drugs are known to induce mitotic catastrophe in cancer cells in their mechanisms of action. Examples of such drugs include bleomycin, doxorubicin, cisplatin, etoposide, and *Taxol*. Anticancer drugs that induce mitotic catastrophe are important for many reasons, some of which are as follows: (i) they are required in lower doses compared to those that induce apoptosis, and (ii) they are efficient against apoptosis-resistant cancer cells due to the fact that mitotic catastrophe occurs in cells with functionally defective apoptosis pathways. Moreover, mitotic catastrophe potentially occurs in p53-deficient and/or cells with a weakened G2/M checkpoint making it relatively selective upon cancer cells. Following the mitotic catastrophe, cells either proceed to necroptosis or apoptosis. In brief, mitotic catastrophe could be considered a mechanism by mechanism by which cells perceive potentially pathogenic genetic instability and respond through oncosuppressive events that lead either to necrosis/necroptosis or apoptosis. Therefore, a better understanding of the molecular patterns of mitotic catastrophe would be of great benefit for the design of anticancer therapeutics [101–106].

#### Senescence

Tissue’s homeostasis involves control of appropriate and balanced cell proliferation as hypo-/hyper-proliferation would result in pathological syndromes. Reported experimental in vitro assays by Leonard Hayflick confined the number of cell divisions that a given cell can achieve to an interval of 50 to 70. This is referred to as the “Hayflick limit” above which the cell becomes senescent. Senescence is a healthy and physiological growth arrest that is different from apoptosis because senescent cells could be metabolically active for a long time. It is also different from the differentiated/specialized cell; it is a programmed limit of replicative lifespan to somatic cells. Senescence is characterized by biochemical and morphological changes resulting from cellular physiological functions such as telomere’s shortening following the cell cycle or prematurely induced by environmental factors, namely oxidative stresses damaging DNA and medication. Cellular senescence frequently occurs and at a higher rate with age [107–111].

Senescence is often considered as a tumor-suppressive mechanism. In that, scientific reports support that it is occurring to prevent an overacting oncogene or an underacting tumor suppressor gene. The oncogenic stress induced by the oncogenes mediates a hyper-replicative process that results in oxidative damage to the DNA due to the generation of unsustainable oxidative stress. This acts as a stimulus to an intrinsic response involving Arf/p53/p21; p16/pRb- and DNA-damage response pathways. Similarly, loss of tumor suppressor genes could induce senescence, as illustrated by the loss of Pten and NF1 genes in mouse and human cells. However, the mechanism of senescence causation is yet not fully understood. The nowadays accepted hallmarks of senescent cells have some opposite outcomes. It is believed that senescent cells could be either a failsafe mechanism, i.e., have tumor-suppressive effects if it shortly progresses to apoptosis or pro-inflammatory, or pro-oncogenic effect mediated by the senescence-associated secretory phenotype (SASP) [112–114].

### Tumor metastasis

The high morbidity and mortality of cancer are primarily due to metastasis rather than primary cancer. Very few patients are diagnosed with localized primary cancer at its early stages. Localized solid cancer would be theoretically cured by surgery and local irradiation if diagnosed early. However, diagnosed patients usually present already established metastasized or metastasizing cancer [115, 116].

Metastasis (metastases in plural) stems from Greek word “*meta*” meaning “change” and “*histanai*” meaning “to place, or cause to stand”; thus, literally metastasis means “changing place.” Metastasis medically refers to a multi-step complex process through which cells break away from a primary tumor to travel through the lymphatic system or bloodstream, colonize a unique microenvironment, and establish malignant growth. Metastasis is theoretically the ideal and privileged target of therapeutics for control and curative treatment of cancer as it is the key feature determining cancer’s malignancy, prognosis, and treatment strategy for any patient. As a general rule: “*all metastasized neoplasms are incontrovertibly malignant*, *but not all neoplasms necessarily metastasize*!” In fact, the probability of cancer to metastasize varies with respect to the type of cancer. For instance, squamous cell carcinoma of the skin has a low probability of metastasizing, while squamous cell carcinoma of the lung metastasis is common. Similarly, metastasis is rarely observed in gliomas. Both squamous cells carcinoma and gliomas are locally invasive and aggressive malignancies that rarely metastasize [117–122].

In general, when cancer metastasizes, the organs colonized are the bones, the brain, the liver, the lymph nodes, and the lungs. To a lesser extent, cancer could also metastasize to the pleural space, abdominal cavity, and other organs like the skin, the muscles, etc.

Although metastasizing cells could travel throughout the body, depending on primary cancer, they tend to target specific organs: (i) lung cancer usually metastasizes to the brain, the bones, liver, and the adrenal glands; (ii) colorectal cancer metastatic cells target preferably to the liver and the lungs; (iii) metastatic breast cancer tends to colonize the bones, the brain, the liver, the lungs, the chest wall, and the brain; and (iv) prostate cancers mostly metastasize to the bones [123–126].

However, metastasis is a long-term (lasts for years or even decades), but discrete complex series of events through which only a few cancer cells would be fruitful in colonizing new sites on distant organs from the original, i.e., primary tumors establish then eventually travel malignant growth (secondary tumor) that is always of the same type, but that disturbs the hosting organ’s physiology. Since depending on the primary tumor, metastatic cells tend to target specific sites, understanding the mechanistic features that drive the motion of cancer cells toward specific organs, the cancer cells-host cell interactions, and the molecular events that favor growth of the cancer cells on a secondary site would be constructive for any eventual promising approach to cancer therapy. To properly establish, metastasis takes three concrete steps, namely (i) intravasation, i.e., cancer cells break off from a primary tumor and enter the circulatory system that could be either the bloodstream ( hematogenous metastasis), the lymphatic system (lymphatic metastasis), or through body cavities (transcoelomic); (ii) the survival to the trauma caused by the intravasation process and survival to the immune system’s attack along with the circulatory system, and (iii) extravasation or the exit from the circulatory system and establishment of malignant growth at the secondary site s[125, 127].

### Genetics of cancer: oncogenes, proto-oncogenes, and tumor suppressor genes

Cancer would be characterized by anarchic cell proliferation and resistance to programmed cell death or apoptosis at the cell level. At the gene level, three events could say to characterize any cancer, namely (i) actively expressed oncogene(s); (ii) inefficient/defective DNA repair genes; and (iii) defective tumor suppressor genes (TSGs). Most of the time, defective DNA repair genes and defective TSGs are taken under the scope of TSGs. These characteristic events could act in concert among and/or between themselves to cause a normal cell to exhibit cancerous properties. Functional mutations broadly fall into three categories, namely mutational inactivation, mutational activation, and mutational gain of function. The mutational inactivation usually occurs in tumor suppressor genes and could be either amorphic, hypomorphic, or antimorphic. The mutational activation of genes could be exemplified by oncogene activation through hypermorphic mutation. Lastly, when a gene is mutated to gain novel function(s), the mutation is called neomorphic [125, 127].

#### Oncogenes and proto-oncogenes

The term oncogene was first introduced by George Todaro & Robert Heubner in 1969 as a derivative from the Greek words “*onkos*” meaning “mass/ bulk,” and “*gignere*/*genui*” (i.e., “gene”), meaning “beget/give birth.” Oncogene refers to any gene that bears the potential to stimulate anarchic cell growth. Experimental approaches, i.e., in vitro/in vivo transfection of normal cells/nude mice with activated oncogenes, would confer a normal cell with activated tumorigenic phenotype/form tumor in the nude mice. However, activating a single oncogene is insufficient to cause healthy cells to exhibit cancer cell properties. In fact, oncogene cooperation is required for active tumorigenesis, i.e., the oncoprotein resulting from the expression of the oncogene would eventually require subsequent synergetic oncomutation/activation of another oncogene/defective tumor suppressor gene or other environmental cues such as viral infection. Thus oncogene cooperation lies in line with the clonal evolution model of cancer and as well as cancer stem cells model [128–133].

Therefrom, oncogenes scope out the functional aspects of genes, that when abnormally overexpressed/gain constitutive expression would eventually contribute to tumorigenesis. Proto-oncogenes mediate cell growth in healthy cells. They are somehow like precursor genes to oncogenes. However, they are often abusively referred to as oncogenes. When proto-oncogenes undergo oncomutation(s), they would eventually convert to oncogenes. The oncomutation, in some cases, involves a change in the gene sequence of a proto-oncogene that results in their constitutive expression. In general, oncomutation is of three types, namely activating mutation (e.g., *K*-*ras*, *H*-*ras*, *N*-*ras*; EGFR), gene amplification (e.g., ErbB2/HER2; *Myc*), and insertion of influential (viral) promoters [134, 135].

It is noteworthy to mention that oncogenes have been first identified in RNA viruses, i.e., retroviruses. Since then, many RNA viruses have been proven to be associated with cancer in both animals and humans. Although oncogenes’ possession is not very common across all retroviruses, the few ones that possess it (them) are highly oncogenic upon infection to animals and human beings. Some of the most known oncogenic viruses include Merkel cell-polyomavirus, Hepatitis B virus, Hepatitis C virus, Epstein Barr virus (EBV), Kaposi’s sarcoma-associated herpesvirus (KSHV), and T-lymphotropic virus. Since the 1970s, many oncogenes have been identified in human cancers. Their occurrence across the cancer types varies from one cancer (sub)-type to another. To exemplify that *k-ras* on computation, a mutation in the gene sequence is prevalent in lung adenocarcinoma and non-small cell lung carcinomas but rarely occurs in small cell lung carcinomas. Another example, this time that manifests as overexpression of an oncogene, is myc gene overexpression. This is seen in both small cell and non-small cells lung carcinomas [51, 136–141].

#### Tumor suppressor genes

Tumor suppressor genes, also called anti-oncogenes, are those genes that functionally mediate in vivo homeostatic cell growth, differentiation, and motility potential. They act either directly or indirectly to slow down cell proliferation, promote DNA repair, and favor apoptosis when required. Any mutation of all kinds that eventually alters these genes’ function would be a step on the path to cancer to the cell. There are broadly four mechanisms that would result in functionally defective tumor suppressor genes: (i) direct mutation/gene silencing mutation; (ii) gene deletion; (iii) loss of heterozygosity; and (iv) promoter hypermethylation [142–146].

### Epigenetic changes in cancer

Epigenetics is mostly concerned with the mechanistic processes that initiate and/or maintain heritably, but reversible alteration (s) in gene regulation in response to environmental stimuli independently of the DNA sequence (that is one of the reasons it is mostly reported as associated to CSC). The epigenetic changes are called epimutations. Subsequent to their initiating signal’s disappearance, they could either disappear (changing back to the state prior to the signal) or last for long (sometimes forever). Epimutations include DNA methylation, histone modification patterns, chromatin remodeling, non-coding RNAs (miRNA and others), polycomb-group of proteins, etc*.* Epigenetic dysregulation could promote carcinogenesis and metastasis. All the aforementioned epimutations have been, in one way or another, reported as clinically significant in the etiology, diagnosis, and/or prognostic outcome of cancer [147–150].

#### Epigenetic modulators, modifiers, and mediators

Genes involved in epigenetic changes are classified into three (3) major classes: epigenetic mediators, epigenetic modifiers, and epigenetic modulators. Epigenetic mediators are peptides/proteins responsible for cancer cells’ stemness character, flexibility, and eventual intratumoral heterogeneity of cancerous tissues. They interact with epigenetic modifiers (HDAC, HAT, HMTs). The later class sets bases for epimutation through DNA methylation, chromatin modulation, histone modification, etc*.* The third and last class, i.e., the epigenetic modulators, are tissues-specific proteins vital to normal cellular functions as they lead the cell and tissue differentiation mechanisms. They transmit environmental signals to the epigenetic modifiers [151–154]

#### DNA methylation

DNA methylation is one of the commonest studied and well-characterized among the epigenetic changes. The process involves transferring a methyl group to position 5′ of the pyrimidine-cytosine of 5′-CpG-3′-rich DNA regions called CpG islands: a palindromic set of cytosine-phosphate-guanine dinucleotides found in the promoter region and other genomic regions. The latter could be a hemimethylated or homo-methylated respectively when on cytosine residues of only one strand or both strands of the DNA are methylated. Methylation could also occur-but to a lesser extent on the cytosine of 5′-CT-3′ or 5′-CA-3′ residues. DNA methylation implies the dissociation of the DNA from transcription factors necessary for activity to the methylating enzymes called DNA-methyltransferases. Some repressor proteins have an affinity to methylated DNA, including MBD2, MBD3, MeCP2, etc*.* DNA methylation thus controls the transcriptional plasticity of a cell. Different variants of DNA-methylation have been reported as etiologic events harboring carcinogenesis and/or metastasis. Among these are aberrant DNA hypomethylation (overexpression of oncogenic proteins; loss of imprinting), aberrant hypermethylation (silencing of TSG), and genomic imprinting (the expression of some genes depends on the parent-of-origin) [155–157].

#### Histone modifications

In eukaryotes, DNA is primarily wrapped around (gene)-regulatory proteins called histones. A nucleosome structurally comprises 146 base pairs (bp) of DNA (approximately two turns) coiled around a pair of H2A, H2B, H3, and H4 histones. H1, the histone linker, is used as an anchor to stabilize some (but not all), nucleosomes. The location and direction of DNA wrapping determine the status of heterochromatin and euchromatin, thus playing a role in both packaging DNA within the nucleus and regulating gene expression. The N-terminal tails of the core canonical histones protrude from the nucleosomal particles and are sometimes subjected to covalent post-translational modifications including methylation, acetylation, phosphorylation, sumoylation, ubiquitinylation, etc. All together, these modification patterns are referred to as “histone code” which is one of the control mechanisms of gene expression through alteration of chromatin structure. The modifications prevent the transcriptional machinery from accessing DNA. They mostly occur on specific amino acids (e.g., methylation (Lys & Arg), acetylation (Lys), phosphorylation (Ser & Thr), ubiquitination and sumoylation (Lys), ADP-ribosylation (Glu)) [147, 158, 159]. Aberrant histone modifications and beyond, i.e., oncohistones, have been reported as associated with various cancer.

#### Chromatin remodeling in cancer

In eukaryotic cells, the nuclear DNA is highly coiled and kinked around histones forming nucleosomes (repeating structural units of chromatin). The later process is for efficiently packaging meters of DNA in a nuclear cell compartment (active, inactive states of chromatin) and a primary level of regulation to gene expression (limits access to gene promoter and enhancer regions). Euchromatin and heterochromatin are hallmarked by epigenetic signatures on DNA and histones that allow their structural organization. The heterochromatin (facultative and constitutive) constitutes the tightly compacted and transcriptionally silent genomic regions, while the euchromatin refers to the transcriptionally active and lightly packed genomic regions. The RNA polymerase and transcriptional factors need to access genes for their eventual expression to take place: tightly coiled genes in chromatin are not accessible. To permit access for the transcription machinery (RNA polymerase, transcription factors…), in eukaryotic cells, the chromatin “*opens*,” changing from its default state, hereby exposing selective genes for expression. This dynamic complex “*opening*” process that modulates the architecture of chromatin to expose genomic DNA is called chromatin remodeling, and it is of vital importance to the functional homeostasis of eukaryotic cells. The process involves various multiprotein complexes called chromatin remodelers that use ATP hydrolysis to mobilize and restructuration of nucleosomes. Thus remodelers play a canonical role in exposing DNA for transcription, chromatin structure (default), DNA-repair mechanism, etc*.* In general, access to nucleosomal DNA is made possible by two (2) major classes of multiprotein complexes, namely (i) covalent histone-modifying complexes (HATs, HDACs, etc*.*), and (ii) ATP-dependent chromatin remodeling complexes for moving, ejecting, and restructuring nucleosomes. CRCs refer to four structurally and functionally distinguishable families in eukaryotes: SWI/SNF, ISWI, CHD, and INO80. Histone modification could favor chromatin “*opening*,” thereby giving ways to a selective portion of DNA, which in turn recruit RNA polymerase II. Various histone modifications have been correlated to chromatin activation [149, 151, 160, 161].

### Epigenetic modulations, nutrients, bioactive food compounds, microbiota, and cancer

Defining cancer (etiology, diagnosis, and/or prognostic) is not an easy task and sometimes depends on the approach-(es) used: it is a cellular disease, a genetic disease, a metabolic disease, a chronic inflammatory disease, an epigenetic (nutritional and/or not), a dysbiosis, etc*.* These are not necessarily meaning different things. In fact, it is a disease that is so complex that a good attempt to define it would merely with a multidisciplinary approach [162–165].

A plethora of scientific reports suggested, based on intriguing evidence, that dietary components (nutrients, bioactive compounds), their interaction with the gut microbiota, could impact, through epigenetic modulations, the pathways involved in gene expression. Although a lot is yet to be discovered about the details of the mechanistic interaction between nutrients/nutrients-microbiota and the epigenetic events leading to pathogenesis (especially tumorigenesis), deepening research fields aiming to understand these mechanisms would be a great advance to cancer research toward a novel therapeutic strategy.

Nutrients and other food components could modulate their metabolic pathways and sometimes reshape the epigenome. They could mediate the phenotypic plasticity of an organism through epigenetic mechanisms, namely DNA methylation, histone modification, chromatin remodeling, etc*.* Therefrom, nutrients and other food components could be said to play an important role in cancer prevention, etiology, development, management, and outcomes. The folate (B_9_), choline, betaine, and vitamin B_12_ are among the most studied one-carbon nutrients, which donate a methyl group to *S-adenosylhomocystein*, converting it to *S-adenosylmethionine* (the universal methyl donor). The latter inhibits methyl-transferases that are responsible for DNA methylation and histone tails methylations. The over-take, normo-take, or insufficient nutrition of one carbon donors have impacts on both gene expression (through epimutations) and the generation of epigenetic signatures to ensure the heritability of gene expression patterns. As such, it is intimately linked to gene expression patterns in developmental physiology and pathological conditions like cancer. In cancer, the highly proliferative cells require these micronutrients to feed their DNA synthesis, methylations, and redox metabolisms [166–168].

In both plants and animals, HATs/HDACs play a crucial role in transferring a functional acetoxy—a group from one organic molecule (donor) to another (acceptor). Acetylation and deacetylation are merely a replacement of an H-atom (in the accepting chemical compound), by an acetoxy group (from the donor), and the removal of an acetoxy group from an organic molecule for an H-atom substitute, respectively. Acetylation usually occurs on the ɛ-amino group of lysine residues of histone tails, dissociating them locally from their nucleosomes and freeing access to DNA to transcriptional machinery, thus promotes gene expression. Conversely, deacetylation reinforces histone-tails-nucleosomal interactions that secure local DNA from transcription. In animals, various physiological functions require reactions ranging from detoxifying xenobiotics to gene regulation via histone modifications. There is an intriguing disparity in the expression and activity of HATs in normal and cancer cells. Some oncogene’s expression during carcinogenesis and metastasis have been reported as associated with HATs overexpression and increased activity (P300/CBP). Conversely, a high expression of HDACs and activity (usually pairing with low HATs expression and activity) would consequentially silence expression of tumor suppressors, thereby promoting progression of tumorigenesis and metastasis [169–171].

A growing body of evidence suggests that some dietary components (found in fruits, cruciferous plants, whole grains, specific micronutrients, dietary fibers, etc*.*) have modulatory effects on histones. In fact, the dietary supply of acetyl group donors would modify the intracellular/nuclear pool of acetate that in turn mediate histone acetylation/deacetylation. A high nuclear pool of acetate would promote acetylation. Like other epigenetic modulation mechanisms, histone modifications starts right from embryogenesis and dynamically continue/reshape throughout the lifespan; thus besides environmental stimuli, it could be influenced by mother’s diet right in the womb and reversibly continues [172–176].

The microbiota refers to communities of commensal microorganisms (≈ 2 kg), living within animals’ gastrointestinal tract. It has been reported that the qualitative and quantitative members of this microbial flora play a very important role in homeostasis through their interactions with their host. The conditions whereby a critical balance of the desired floral community has been established is known as eubiosis and is expected/reported to be associated with health conditions. In contrast, dysbiosis (i.e., an unbalanced ratio of desired: undesired community members) is associated to various disease conditions. Dietary components influence the diversity and function of the microbial community, impacting the thickness of the mucus (separating them from), protecting the intestine. Eubiosis is usually reflected by thick mucus size, while dysbiosis is associated to thin mucus size [177–179]. Through their metabolisms (of dietary fibers, bioactive compounds components of food, short-chain fatty acids, etc*.*), and in concert with environmental cues, they produce bioactive compounds that could initiate or promote epigenetic modulations and/or set epigenetic signatures. In concert with the energy balance, the dietary composition and abundance dictate the availability of certain nutrients, which are cofactors to enzymes involved in epigenetic events. In other words, they produced metabolites that could interact or provide substrates for methyl-transferase (DNA, and/or histone methylation/demethylation), HATs/HDACs for histone acetylation/deacetylation, etc*.* Various bacterial *species* among the members of the gut microbiota, depending on the microenvironmental conditions, have been proven to produce oncometabolite, immunomodulatory, “*onco*-*reppressive*,” or *oncolytic* metabolites that in turn mediate ubiquitous epigenetic mechanisms [180–186].

## Conclusions

A full comprehension of cancer right from its initiation through metastasis would require a multidisciplinary approach that would eventually reveal its molecular etiology and sets bases forestalling its molecular pathology. Progresses in cancer research mediate the breaking of frontiers of many scientific disciplines down to a huge body of interdisciplinary approaches wherein overlap life sciences for a better understanding of the wishful organismal harmonic states, their maintenance and their otherwise disturbances that would potentiate carcinogenesis.

Molecular genetics elucidate that genetic defects could, when they occur on genes of cell cycle checkpoints, eventually potentiate unrestricted cell proliferation or oncometabolisms when a gene that encodes for a crucial metabolic enzyme is defective. Conversely, CSC and CSCP models broke the myth of evolutionary carcinogenesis by elucidating theoretical mechanisms hitherto unsuspected. Moreover, the epigenetic mechanisms involved in cancer cells have been linked to the dietary and energy balance which in turn harbor the dynamic community and metabolism of the gut microbiota (production of antioxydants, anti-cancer, oncometabolites, onco-suppressive metabolites, etc.). Knowledge of these multiple facets of cancer’s molecular etiology and pathology would better serve for drug design of an efficient and effective personalized medicine.

## Data Availability

Not applicable.

## Abbreviations

Arf: ADP-ribosylation factor
CAF: Cancer-associated fibroblasts
CDH: Cadherin
CDK: Cyclin-dependent-kinase
CIP: CDK interacting protein
CKI: Cyclin-dependent-kinase-inhibitors
CpG: Cytosine-phosphate-guanine dinucleotides
CSC: Cancer stem cell
CSCP: Cancer stem cell plasticity
CSFs: Colony-stimulating factors
DMP1: Dentin matrix acidic phosphoprotein 1
DNA: Deoxyribonucleic acid
E2F: A group of genes that code for transcription factor
EBV: Epstein-barr virus
ECM: Extracellular matrix
EGF: Epidermal growth factor
EMT: Epithelial-mesenchymal transition
EMT-TF: Epithelial-mesenchymal transition transition factor
ErbB2: Erythroblastic oncogene B
FOXM1C: Forkhead box protein M
FOXO: Forkhead box transcription factors
GTP: Guanosine triphosphate
HATs: Histone acetyltransferases
HDAC: Histone deacetylase
HER2: Human epidermal growth factor
HGF: Hepatocyte growth factor
INK: Inhibitor of CDK (cyclin dependent kinase inhibitor)
ISWI: Imitation SWI
JUN: *ju-nana* (proto-oncogene)
*k-ras*: *kristen rat sarcoma* (viral oncogene)
KSHV: Kaposi’s sarcoma-associated herpes virus
MBD2: Methyl-CpG binding domain protein 2
MeCP: MECP2 (methyl-CpG binding protein 2)
MET: Mesenchymal-epithelial transition
miRNA: microRNA
MMPs: Matrix metallopeptidase/proteinases
MYC: MYeloCytomatosis (proto-oncogene)
NF1: Neurofibromin 1
NFR1: Nuclear respiratory factor1
PDGF: Platelet-derived growth factor
pRb: Retinoblastoma protein
Pten: Phosphatase and tensin homolog (deleted on chromosome 10)
*Ras*: A family of proteins (GPTases) responsible for cell proliferation
Rb: Retinoblastoma protein
SASP: Senescence-associated secretory phenotype
SNAIL/ SLUG: SWItch/sucrose non-fermentable
TGF-β: Transforming growth factor beta
TNF-α: Tumor necrosis factor α
TSG: Tumor suppressor genes
ZEB1: Zinc finger E-box

## References

[1] Baker S, Silins I, Guo Y, Ali I, Hogberg J, Stenius U, et al. Automatic semantic classification of scientific literature according to the hallmarks of cancer. Bioinformatics. 2016;32(3):432–40. 10.1093/bioinformatics/btv585. 26454282 10.1093/bioinformatics/btv585

[2] Cai Q, Yuan Z. Overview of ınfectious causes of human cancers. Adv Exp Med Biol. 2017;1018:1–9. 10.1007/978-981-10-5765-6_1. 29052128 10.1007/978-981-10-5765-6_1

[3] De Flora S, La Maestra S. Epidemiology of cancers of infectious origin and prevention strategies. J Prev Med Hyg. 2015;56(1):E15–20. 26789827 PMC4718340

[4] Moore PS, Chang Y. Why do viruses cause cancer? Highlights of the first century of human tumour virology. Nat Rev Cancer. 2010;10(12):878–89. 10.1038/nrc2961. 21102637 10.1038/nrc2961 PMC3718018

[5] Hong SN. Genetic and epigenetic alterations of colorectal cancer. Intest Res. 2018;16(3):327–37. 10.5217/ir.2018.16.3.327. 30090031 10.5217/ir.2018.16.3.327 PMC6077299

[6] Puneet KHR. Kumari S, Tiwari S, Khanna A, Narayan G. Epigenetic mechanisms and events in gastric cancer-emerging novel biomarkers. Pathol Oncol Res. 2018;24(4):757–70. 10.1007/s12253-018-0410-z. 29552712 10.1007/s12253-018-0410-z

[7] Hanahan D, Weinberg RA. Hallmarks of cancer: the next generation. Cell. 2011;144(5):646–74. 10.1016/j.cell.2011.02.013. 21376230 10.1016/j.cell.2011.02.013

[8] Moses C, Garcia-Bloj B, Harvey AR, Blancafort P. Hallmarks of cancer: the CRISPR generation. Eur J Cancer. 2018;93:10–8. 10.1016/j.ejca.2018.01.002. 29433054 10.1016/j.ejca.2018.01.002

[9] Bottcher M, Baur R, Stoll A, Mackensen A, Mougiakakos D. Linking ımmunoevasion and metabolic reprogramming in B-cell-derived lymphomas. Front Oncol. 2020;10:594782. 10.3389/fonc.2020.594782. 33251150 10.3389/fonc.2020.594782 PMC7674840

[10] Barnum KJ, O'Connell MJ. Cell cycle regulation by checkpoints. Methods Mol Biol. 2014;1170:29–40. 10.1007/978-1-4939-0888-2_2. 24906307 10.1007/978-1-4939-0888-2_2 PMC4990352

[11] Elledge SJ. Cell cycle checkpoints: preventing an identity crisis. Science. 1996;274(5293):1664–72. 10.1126/science.274.5293.1664. 8939848 10.1126/science.274.5293.1664

[12] Kastan MB, Bartek J. Cell-cycle checkpoints and cancer. Nature. 2004;432(7015):316–23. 10.1038/nature03097. 15549093 10.1038/nature03097

[13] Lukas J, Lukas C, Bartek J. Mammalian cell cycle checkpoints: signalling pathways and their organization in space and time. DNA Repair (Amst). 2004;3(8-9):997–1007. 10.1016/j.dnarep.2004.03.006. 15279786 10.1016/j.dnarep.2004.03.006

[14] Hydbring P, Malumbres M, Sicinski P. Non-canonical functions of cell cycle cyclins and cyclin-dependent kinases. Nat Rev Mol Cell Biol. 2016;17(5):280–92. 10.1038/nrm.2016.27. 27033256 10.1038/nrm.2016.27 PMC4841706

[15] Houtgraaf JH, Versmissen J, van der Giessen WJ. A concise review of DNA damage checkpoints and repair in mammalian cells. Cardiovasc Revasc Med. 2006;7(3):165–72. 10.1016/j.carrev.2006.02.002. 16945824 10.1016/j.carrev.2006.02.002

[16] Johnson DG, Walker CL. Cyclins and cell cycle checkpoints. Annu Rev Pharmacol Toxicol. 1999;39(1):295–312. 10.1146/annurev.pharmtox.39.1.295. 10331086 10.1146/annurev.pharmtox.39.1.295

[17] Bertoni G. Cell cycle regulation by chlamydomonas cyclin-dependent protein kinases. Plant Cell. 2018;30(2):271. 10.1105/tpc.18.00103. 29437987 10.1105/tpc.18.00103 PMC5868689

[18] Ekholm SV, Reed SI. Regulation of G(1) cyclin-dependent kinases in the mammalian cell cycle. Curr Opin Cell Biol. 2000;12(6):676–84. 10.1016/s0955-0674(00)00151-4. 11063931 10.1016/s0955-0674(00)00151-4

[19] Morgan DO. Principles of CDK regulation. Nature. 1995;374(6518):131–4. 10.1038/374131a0. 7877684 10.1038/374131a0

[20] Vermeulen K, Van Bockstaele DR, Berneman ZN. The cell cycle: a review of regulation, deregulation and therapeutic targets in cancer. Cell Prolif. 2003;36(3):131–49. 10.1046/j.1365-2184.2003.00266.x. 12814430 10.1046/j.1365-2184.2003.00266.x PMC6496723

[21] Canepa ET, Scassa ME, Ceruti JM, Marazita MC, Carcagno AL, Sirkin PF, et al. INK4 proteins, a family of mammalian CDK inhibitors with novel biological functions. IUBMB Life. 2007;59(7):419–26. 10.1080/15216540701488358. 17654117 10.1080/15216540701488358

[22] Corbet C. Stem cell metabolism in cancer and healthy tissues: pyruvate in the limelight. Front Pharmacol. 2017;8:958. 10.3389/fphar.2017.00958. 29403375 10.3389/fphar.2017.00958 PMC5777397

[23] Denicourt C, Dowdy SF. Cip/Kip proteins: more than just CDKs inhibitors. Genes Dev. 2004;18(8):851–5. 10.1101/gad.1205304. 15107401 10.1101/gad.1205304

[24] Harper JW, Elledge SJ, Keyomarsi K, Dynlacht B, Tsai LH, Zhang P, et al. Inhibition of cyclin-dependent kinases by p21. Mol Biol Cell. 1995;6(4):387–400. 10.1091/mbc.6.4.387. 7626805 10.1091/mbc.6.4.387 PMC301199

[25] Reynisdottir I, Polyak K, Iavarone A, Massague J. Kip/Cip and Ink4 Cdk inhibitors cooperate to induce cell cycle arrest in response to TGF-beta. Genes Dev. 1995;9(15):1831–45. 10.1101/gad.9.15.1831. 7649471 10.1101/gad.9.15.1831

[26] Quereda V, Porlan E, Canamero M, Dubus P, Malumbres M. An essential role for Ink4 and Cip/Kip cell-cycle inhibitors in preventing replicative stress. Cell Death Differ. 2016;23(3):430–41. 10.1038/cdd.2015.112. 26292757 10.1038/cdd.2015.112 PMC5072439

[27] Morgunova E, Yin Y, Jolma A, Dave K, Schmierer B, Popov A, et al. Structural insights into the DNA-binding specificity of E2F family transcription factors. Nat Commun. 2015;6(1):10050. 10.1038/ncomms10050. 26632596 10.1038/ncomms10050 PMC4686757

[28] Ren B, Cam H, Takahashi Y, Volkert T, Terragni J, Young RA, et al. E2F integrates cell cycle progression with DNA repair, replication, and G(2)/M checkpoints. Genes Dev. 2002;16(2):245–56. 10.1101/gad.949802. 11799067 10.1101/gad.949802 PMC155321

[29] Schulze A, Zerfass K, Spitkovsky D, Middendorp S, Berges J, Helin K, et al. Cell cycle regulation of the cyclin A gene promoter is mediated by a variant E2F site. Proc Natl Acad Sci U S A. 1995;92(24):11264–8. 10.1073/pnas.92.24.11264. 7479977 10.1073/pnas.92.24.11264 PMC40612

[30] Thwaites MJ, Cecchini MJ, Passos DT, Welch I, Dick FA. Interchangeable roles for E2F transcriptional repression by the retinoblastoma protein and p27KIP1-cyclin-dependent kinase regulation in cell cycle control and tumor suppression. Mol Cell Biol. 2017;37(2). 10.1128/MCB.00561-16. 10.1128/MCB.00561-16 PMC5214858 27821477

[31] Bendris N, Lemmers B, Blanchard JM. Cell cycle, cytoskeleton dynamics and beyond: the many functions of cyclins and CDK inhibitors. Cell Cycle. 2015;14(12):1786–98. 10.1080/15384101.2014.998085. 25789852 10.1080/15384101.2014.998085 PMC4614797

[32] Blanchet E, Annicotte JS, Lagarrigue S, Aguilar V, Clape C, Chavey C, et al. E2F transcription factor-1 regulates oxidative metabolism. Nat Cell Biol. 2011;13(9):1146–52. 10.1038/ncb2309. 21841792 10.1038/ncb2309 PMC3849758

[33] Fajas L, Lopez-Mejia IC. CDK4, a new metabolic sensor that antagonizes AMPK. Mol Cell Oncol. 2018;5(5):e1409862. 10.1080/23723556.2017.1409862. 30263936 10.1080/23723556.2017.1409862 PMC6154832

[34] Karimian A, Ahmadi Y, Yousefi B. Multiple functions of p21 in cell cycle, apoptosis and transcriptional regulation after DNA damage. DNA Repair (Amst). 2016;42:63–71. 10.1016/j.dnarep.2016.04.008. 27156098 10.1016/j.dnarep.2016.04.008

[35] Lopez-Mejia IC, Fajas L. Cell cycle regulation of mitochondrial function. Curr Opin Cell Biol. 2015;33:19–25. 10.1016/j.ceb.2014.10.006. 25463842 10.1016/j.ceb.2014.10.006

[36] Wang Z, Fan M, Candas D, Zhang TQ, Qin L, Eldridge A, et al. Cyclin B1/Cdk1 coordinates mitochondrial respiration for cell-cycle G2/M progression. Dev Cell. 2014;29(2):217–32. 10.1016/j.devcel.2014.03.012. 24746669 10.1016/j.devcel.2014.03.012 PMC4156313

[37] Asghar U, Witkiewicz AK, Turner NC, Knudsen ES. The history and future of targeting cyclin-dependent kinases in cancer therapy. Nat Rev Drug Discov. 2015;14(2):130–46. 10.1038/nrd4504. 25633797 10.1038/nrd4504 PMC4480421

[38] Musgrove EA, Caldon CE, Barraclough J, Stone A, Sutherland RL. Cyclin D as a therapeutic target in cancer. Nat Rev Cancer. 2011;11(8):558–72. 10.1038/nrc3090. 21734724 10.1038/nrc3090

[39] Otto T, Sicinski P. Cell cycle proteins as promising targets in cancer therapy. Nat Rev Cancer. 2017;17(2):93–115. 10.1038/nrc.2016.138. 28127048 10.1038/nrc.2016.138 PMC5345933

[40] Jayachandran A, Dhungel B, Steel JC. Epithelial-to-mesenchymal plasticity of cancer stem cells: therapeutic targets in hepatocellular carcinoma. J Hematol Oncol. 2016;9(1):74. 10.1186/s13045-016-0307-9. 27578206 10.1186/s13045-016-0307-9 PMC5006452

[41] Marjanovic ND, Weinberg RA, Chaffer CL. Cell plasticity and heterogeneity in cancer. Clin Chem. 2013;59(1):168–79. 10.1373/clinchem.2012.184655. 23220226 10.1373/clinchem.2012.184655 PMC6220421

[42] Abou-Antoun TJ, Hale JS, Lathia JD, Dombrowski SM. Brain cancer stem cells in adults and children: cell biology and therapeutic ımplications. Neurotherapeutics. 2017;14(2):372–84. 10.1007/s13311-017-0524-0. 28374184 10.1007/s13311-017-0524-0 PMC5398995

[43] Ajani JA, Song S, Hochster HS, Steinberg IB. Cancer stem cells: the promise and the potential. Semin Oncol. 2015;42(Suppl 1):S3–17. 10.1053/j.seminoncol.2015.01.001. 25839664 10.1053/j.seminoncol.2015.01.001

[44] O'Connor ML, Xiang D, Shigdar S, Macdonald J, Li Y, Wang T, et al. Cancer stem cells: a contentious hypothesis now moving forward. Cancer Lett. 2014;344(2):180–7. 10.1016/j.canlet.2013.11.012. 24333726 10.1016/j.canlet.2013.11.012

[45] Pattabiraman DR, Weinberg RA. Tackling the cancer stem cells—what challenges do they pose? Nat Rev Drug Discov. 2014;13(7):497–512. 10.1038/nrd4253. 24981363 10.1038/nrd4253 PMC4234172

[46] Bahmad HF, Elajami MK, El Zarif T, Bou-Gharios J, Abou-Antoun T, Abou-Kheir W. Drug repurposing towards targeting cancer stem cells in pediatric brain tumors. Cancer Metastasis Rev. 2020;39(1):127–48. 10.1007/s10555-019-09840-2. 31919619 10.1007/s10555-019-09840-2

[47] Batlle E, Clevers H. Cancer stem cells revisited. Nat Med. 2017;23(10):1124–34. 10.1038/nm.4409. 28985214 10.1038/nm.4409

[48] Yoshida GJ, Saya H. Therapeutic strategies targeting cancer stem cells. Cancer Sci. 2016;107(1):5–11. 10.1111/cas.12817. 26362755 10.1111/cas.12817 PMC4724810

[49] Albini A, Bruno A, Gallo C, Pajardi G, Noonan DM, Dallaglio K. Cancer stem cells and the tumor microenvironment: interplay in tumor heterogeneity. Connect Tissue Res. 2015;56(5):414–25. 10.3109/03008207.2015.1066780. 26291921 10.3109/03008207.2015.1066780 PMC4673538

[50] Balkwill FR, Capasso M, Hagemann T. The tumor microenvironment at a glance. J Cell Sci. 2012;125(Pt 23):5591–6. 10.1242/jcs.116392. 23420197 10.1242/jcs.116392

[51] Lau L, Gray EE, Brunette RL, Stetson DB. DNA tumor virus oncogenes antagonize the cGAS-STING DNA-sensing pathway. Science. 2015;350(6260):568–71. 10.1126/science.aab3291. 26405230 10.1126/science.aab3291 PMC12974531

[52] Mbeunkui F, Johann DJ Jr. Cancer and the tumor microenvironment: a review of an essential relationship. Cancer Chemother Pharmacol. 2009;63(4):571–82. 10.1007/s00280-008-0881-9. 19083000 10.1007/s00280-008-0881-9 PMC2858592

[53] Plaks V, Kong N, Werb Z. The cancer stem cell niche: how essential is the niche in regulating stemness of tumor cells? Cell Stem Cell. 2015;16(3):225–38. 10.1016/j.stem.2015.02.015. 25748930 10.1016/j.stem.2015.02.015 PMC4355577

[54] Andrade SS, Sumikawa JT, Castro ED, Batista FP, Paredes-Gamero E, Oliveira LC, et al. Interface between breast cancer cells and the tumor microenvironment using platelet-rich plasma to promote tumor angiogenesis—influence of platelets and fibrin bundles on the behavior of breast tumor cells. Oncotarget. 2017;8(10):16851–74. 10.18632/oncotarget.15170. 28187434 10.18632/oncotarget.15170 PMC5370006

[55] Guo S, Deng CX. Effect of stromal cells in tumor microenvironment on metastasis ınitiation. Int J Biol Sci. 2018;14(14):2083–93. 10.7150/ijbs.25720. 30585271 10.7150/ijbs.25720 PMC6299363

[56] Tugues S, Ducimetiere L, Friebel E, Becher B. Innate lymphoid cells as regulators of the tumor microenvironment. Semin Immunol. 2019;41:101270. 10.1016/j.smim.2019.03.002. 30871769 10.1016/j.smim.2019.03.002

[57] Makohon-Moore AP, Zhang M, Reiter JG, Bozic I, Allen B, Kundu D, et al. Limited heterogeneity of known driver gene mutations among the metastases of individual patients with pancreatic cancer. Nat Genet. 2017;49(3):358–66. 10.1038/ng.3764. 28092682 10.1038/ng.3764 PMC5663439

[58] Cabrera MC, Hollingsworth RE, Hurt EM. Cancer stem cell plasticity and tumor hierarchy. World J Stem Cells. 2015;7(1):27–36. 10.4252/wjsc.v7.i1.27. 25621103 10.4252/wjsc.v7.i1.27 PMC4300934

[59] Shlush LI, Hershkovitz D. Clonal evolution models of tumor heterogeneity. Am Soc Clin Oncol Educ Book. 2015:e662–5. 10.14694/EdBook_AM.2015.35.e662. 10.14694/EdBook_AM.2015.35.e662 25993239

[60] Scott J, Marusyk A. Somatic clonal evolution: a selection-centric perspective. Biochim Biophys Acta Rev Cancer. 2017;1867(2):139–50. 10.1016/j.bbcan.2017.01.006. 28161395 10.1016/j.bbcan.2017.01.006

[61] Zhao B, Hemann MT, Lauffenburger DA. Modeling tumor clonal evolution for drug combinations design. Trends Cancer. 2016;2(3):144–58. 10.1016/j.trecan.2016.02.001. 28435907 10.1016/j.trecan.2016.02.001 PMC5400294

[62] Campbell SL, Wellen KE. Metabolic signaling to the nucleus in cancer. Mol Cell. 2018;71(3):398–408. 10.1016/j.molcel.2018.07.015. 30075141 10.1016/j.molcel.2018.07.015

[63] Badve S, Nakshatri H. Breast-cancer stem cells-beyond semantics. Lancet Oncol. 2012;13(1):e43–8. 10.1016/S1470-2045(11)70191-7. 22225725 10.1016/S1470-2045(11)70191-7

[64] Rich JN. Cancer stem cells: understanding tumor hierarchy and heterogeneity. Medicine (Baltimore). 2016;95(1 Suppl 1):S2–7. 10.1097/MD.0000000000004764. 27611934 10.1097/MD.0000000000004764 PMC5599210

[65] Shackleton M, Quintana E, Fearon ER, Morrison SJ. Heterogeneity in cancer: cancer stem cells versus clonal evolution. Cell. 2009;138(5):822–9. 10.1016/j.cell.2009.08.017. 19737509 10.1016/j.cell.2009.08.017

[66] Hay ED. An overview of epithelio-mesenchymal transformation. Acta Anat (Basel). 1995;154(1):8–20. 10.1159/000147748. 8714286 10.1159/000147748

[67] Kiefer JC, Nieto A, Thiery JP. Primer and interview: epithelial to mesenchymal transition. [Interview by Julie Kiefer]. Dev Dyn. 2008;237(10):2769–74. 10.1002/dvdy.21696. 18816863 10.1002/dvdy.21696

[68] Zavadil J, Haley J, Kalluri R, Muthuswamy SK, Thompson E. Epithelial-mesenchymal transition. Cancer Res. 2008;68(23):9574–7. 10.1158/0008-5472.CAN-08-2316. 19047131 10.1158/0008-5472.CAN-08-2316

[69] Kalluri R. EMT: when epithelial cells decide to become mesenchymal-like cells. J Clin Invest. 2009;119(6):1417–9. 10.1172/JCI39675. 19487817 10.1172/JCI39675 PMC2689122

[70] Kalluri R, Weinberg RA. The basics of epithelial-mesenchymal transition. J Clin Invest. 2009;119(6):1420–8. 10.1172/JCI39104. 19487818 10.1172/JCI39104 PMC2689101

[71] Zeisberg M, Neilson EG. Biomarkers for epithelial-mesenchymal transitions. J Clin Invest. 2009;119(6):1429–37. 10.1172/JCI36183. 19487819 10.1172/JCI36183 PMC2689132

[72] Sohal SS, Mahmood MQ, Walters EH. Clinical significance of epithelial mesenchymal transition (EMT) in chronic obstructive pulmonary disease (COPD): potential target for prevention of airway fibrosis and lung cancer. Clin Transl Med. 2014;3(1):33. 10.1186/s40169-014-0033-2. 26932377 10.1186/s40169-014-0033-2 PMC4607924

[73] Nistico P, Bissell MJ, Radisky DC. Epithelial-mesenchymal transition: general principles and pathological relevance with special emphasis on the role of matrix metalloproteinases. Cold Spring Harb Perspect Biol. 2012;4(2). 10.1101/cshperspect.a011908. 10.1101/cshperspect.a011908 PMC3281569 22300978

[74] Nieto MA. Epithelial-Mesenchymal Transitions in development and disease: old views and new perspectives. Int J Dev Biol. 2009;53(8-10):1541–7. 10.1387/ijdb.072410mn. 19247945 10.1387/ijdb.072410mn

[75] Radisky DC. Epithelial-mesenchymal transition. J Cell Sci. 2005;118(Pt 19):4325–6. 10.1242/jcs.02552. 16179603 10.1242/jcs.02552

[76] Son B, Lee S, Youn H, Kim E, Kim W, Youn B. The role of tumor microenvironment in therapeutic resistance. Oncotarget. 2017;8(3):3933–45. 10.18632/oncotarget.13907. 27965469 10.18632/oncotarget.13907 PMC5354804

[77] Campos-da-Paz M, Dorea JG, Galdino AS, Lacava ZGM. de Fatima Menezes Almeida Santos M. Carcinoembryonic antigen (CEA) and hepatic metastasis in colorectal cancer: update on biomarker for clinical and biotechnological approaches. Recent Pat Biotechnol. 2018;12(4):269–79. 10.2174/1872208312666180731104244. 30062978 10.2174/1872208312666180731104244

[78] Zhang J, Yao H, Song G, Liao X, Xian Y, Li W. Regulation of epithelial-mesenchymal transition by tumor-associated macrophages in cancer. Am J Transl Res. 2015;7(10):1699–711. 26692918 PMC4656751

[79] Machesky LM. Lamellipodia and filopodia in metastasis and invasion. FEBS Lett. 2008;582(14):2102–11. 10.1016/j.febslet.2008.03.039. 18396168 10.1016/j.febslet.2008.03.039

[80] Revach OY, Geiger B. The interplay between the proteolytic, invasive, and adhesive domains of invadopodia and their roles in cancer invasion. Cell Adh Migr. 2014;8(3):215–25. 10.4161/cam.27842. 24714132 10.4161/cam.27842 PMC4198345

[81] Weber A, Klocker H, Oberacher H, Gnaiger E, Neuwirt H, Sampson N, et al. Succinate accumulation ıs associated with a shift of mitochondrial respiratory control and HIF-1alpha upregulation in PTEN negative prostate cancer cells. Int J Mol Sci. 2018;19(7). 10.3390/ijms19072129. 10.3390/ijms19072129 PMC6073160 30037119

[82] Bravo-Cordero JJ, Hodgson L, Condeelis J. Directed cell invasion and migration during metastasis. Curr Opin Cell Biol. 2012;24(2):277–83. 10.1016/j.ceb.2011.12.004. 22209238 10.1016/j.ceb.2011.12.004 PMC3320684

[83] Kiesslich T, Pichler M, Neureiter D. Epigenetic control of epithelial-mesenchymal-transition in human cancer. Mol Clin Oncol. 2013;1(1):3–11. 10.3892/mco.2012.28. 24649114 10.3892/mco.2012.28 PMC3956244

[84] Lamouille S, Xu J, Derynck R. Molecular mechanisms of epithelial-mesenchymal transition. Nat Rev Mol Cell Biol. 2014;15(3):178–96. 10.1038/nrm3758. 24556840 10.1038/nrm3758 PMC4240281

[85] Oral D, Erkekoglu P, Kocer-Gumusel B, Chao MW. Epithelial-mesenchymal transition: a special focus on phthalates and bisphenol A. J Environ Pathol Toxicol Oncol. 2016;35(1):43–58. 10.1615/JEnvironPatholToxicolOncol.2016014200. 27279583 10.1615/JEnvironPatholToxicolOncol.2016014200

[86] Serrano-Gomez SJ, Maziveyi M, Alahari SK. Regulation of epithelial-mesenchymal transition through epigenetic and post-translational modifications. Mol Cancer. 2016;15(1):18. 10.1186/s12943-016-0502-x. 26905733 10.1186/s12943-016-0502-x PMC4765192

[87] Gomis RR, Gawrzak S. Tumor cell dormancy. Mol Oncol. 2017;11(1):62–78. 10.1016/j.molonc.2016.09.009. 28017284 10.1016/j.molonc.2016.09.009 PMC5423221

[88] Tsai JH, Yang J. Epithelial-mesenchymal plasticity in carcinoma metastasis. Genes Dev. 2013;27(20):2192–206. 10.1101/gad.225334.113. 24142872 10.1101/gad.225334.113 PMC3814640

[89] Fares J, Fares MY, Khachfe HH, Salhab HA, Fares Y. Molecular principles of metastasis: a hallmark of cancer revisited. Signal Transduct Target Ther. 2020;5(1):28. 10.1038/s41392-020-0134-x. 32296047 10.1038/s41392-020-0134-x PMC7067809

[90] Chaffer CL, San Juan BP, Lim E, Weinberg RA. EMT, cell plasticity and metastasis. Cancer Metastasis Rev. 2016;35(4):645–54. 10.1007/s10555-016-9648-7. 27878502 10.1007/s10555-016-9648-7

[91] Garg M. Epithelial plasticity and cancer stem cells: Major mechanisms of cancer pathogenesis and therapy resistance. World J Stem Cells. 2017;9(8):118–26. 10.4252/wjsc.v9.i8.118. 28928908 10.4252/wjsc.v9.i8.118 PMC5583530

[92] Lee HW, Handlogten ME, Osis G, Clapp WL, Wakefield DN, Verlander JW, et al. Expression of sodium-dependent dicarboxylate transporter 1 (NaDC1/SLC13A2) in normal and neoplastic human kidney. Am J Physiol Renal Physiol. 2017;312(3):F427–F35. 10.1152/ajprenal.00559.2016. 27927654 10.1152/ajprenal.00559.2016 PMC5374311

[93] Neelakantan D, Drasin DJ, Ford HL. Intratumoral heterogeneity: clonal cooperation in epithelial-to-mesenchymal transition and metastasis. Cell Adh Migr. 2015;9(4):265–76. 10.4161/19336918.2014.972761. 25482627 10.4161/19336918.2014.972761 PMC4594578

[94] Schell JC, Olson KA, Jiang L, Hawkins AJ, Van Vranken JG, Xie J, et al. A role for the mitochondrial pyruvate carrier as a repressor of the Warburg effect and colon cancer cell growth. Mol Cell. 2014;56(3):400–13. 10.1016/j.molcel.2014.09.026. 25458841 10.1016/j.molcel.2014.09.026 PMC4268416

[95] Tang XP, Chen Q, Li Y, Wang Y, Zou HB, Fu WJ, et al. Mitochondrial pyruvate carrier 1 functions as a tumor suppressor and predicts the prognosis of human renal cell carcinoma. Lab Invest. 2019;99(2):191–9. 10.1038/s41374-018-0138-0. 30291323 10.1038/s41374-018-0138-0

[96] Castedo M, Perfettini JL, Roumier T, Andreau K, Medema R, Kroemer G. Cell death by mitotic catastrophe: a molecular definition. Oncogene. 2004;23(16):2825–37. 10.1038/sj.onc.1207528. 15077146 10.1038/sj.onc.1207528

[97] Vakifahmetoglu H, Olsson M, Zhivotovsky B. Death through a tragedy: mitotic catastrophe. Cell Death Differ. 2008;15(7):1153–62. 10.1038/cdd.2008.47. 18404154 10.1038/cdd.2008.47

[98] Hayashi MT, Cesare AJ, Fitzpatrick JA, Lazzerini-Denchi E, Karlseder J. A telomere-dependent DNA damage checkpoint induced by prolonged mitotic arrest. Nat Struct Mol Biol. 2012;19(4):387–94. 10.1038/nsmb.2245. 22407014 10.1038/nsmb.2245 PMC3319806

[99] Orth JD, Loewer A, Lahav G, Mitchison TJ. Prolonged mitotic arrest triggers partial activation of apoptosis, resulting in DNA damage and p53 induction. Mol Biol Cell. 2012;23(4):567–76. 10.1091/mbc.E11-09-0781. 22171325 10.1091/mbc.E11-09-0781 PMC3279386

[100] Uetake Y, Sluder G. Prolonged prometaphase blocks daughter cell proliferation despite normal completion of mitosis. Curr Biol. 2010;20(18):1666–71. 10.1016/j.cub.2010.08.018. 20832310 10.1016/j.cub.2010.08.018 PMC2946429

[101] Burns TF, Fei P, Scata KA, Dicker DT, El-Deiry WS. Silencing of the novel p53 target gene Snk/Plk2 leads to mitotic catastrophe in paclitaxel (taxol)-exposed cells. Mol Cell Biol. 2003;23(16):5556–71. 10.1128/mcb.23.16.5556-5571.2003. 12897130 10.1128/MCB.23.16.5556-5571.2003 PMC166320

[102] Maskey D, Yousefi S, Schmid I, Zlobec I, Perren A, Friis R, et al. ATG5 is induced by DNA-damaging agents and promotes mitotic catastrophe independent of autophagy. Nat Commun. 2013;4(1):2130. 10.1038/ncomms3130. 23945651 10.1038/ncomms3130 PMC3753548

[103] Portugal J, Mansilla S, Bataller M. Mechanisms of drug-induced mitotic catastrophe in cancer cells. Curr Pharm Des. 2010;16(1):69–78. 10.2174/138161210789941801. 20214619 10.2174/138161210789941801

[104] Vitale I, Galluzzi L, Castedo M, Kroemer G. Mitotic catastrophe: a mechanism for avoiding genomic instability. Nat Rev Mol Cell Biol. 2011;12(6):385–92. 10.1038/nrm3115. 21527953 10.1038/nrm3115

[105] Woods CM, Zhu J, McQueney PA, Bollag D, Lazarides E. Taxol-induced mitotic block triggers rapid onset of a p53-independent apoptotic pathway. Mol Med. 1995;1(5):506–26. 10.1007/BF03401588. 8529117 PMC2229961

[106] Fujie Y, Yamamoto H, Ngan CY, Takagi A, Hayashi T, Suzuki R, et al. Oxaliplatin, a potent inhibitor of survivin, enhances paclitaxel-induced apoptosis and mitotic catastrophe in colon cancer cells. Jpn J Clin Oncol. 2005;35(8):453–63. 10.1093/jjco/hyi130. 16024531 10.1093/jjco/hyi130

[107] Childs BG, Durik M, Baker DJ, van Deursen JM. Cellular senescence in aging and age-related disease: from mechanisms to therapy. Nat Med. 2015;21(12):1424–35. 10.1038/nm.4000. 26646499 10.1038/nm.4000 PMC4748967

[108] Lidzbarsky G, Gutman D, Shekhidem HA, Sharvit L, Atzmon G. Genomic ınstabilities, cellular senescence, and aging: ın vitro, ın vivo and aging-like human syndromes. Front Med (Lausanne). 2018;5:104. 10.3389/fmed.2018.00104. 29719834 10.3389/fmed.2018.00104 PMC5913290

[109] McHugh D, Gil J. Senescence and aging: Causes, consequences, and therapeutic avenues. J Cell Biol. 2018;217(1):65–77. 10.1083/jcb.201708092. 29114066 10.1083/jcb.201708092 PMC5748990

[110] Tominaga K. The emerging role of senescent cells in tissue homeostasis and pathophysiology. Pathobiol Aging Age Relat Dis. 2015;5(1):27743. 10.3402/pba.v5.27743. 25994420 10.3402/pba.v5.27743 PMC4439419

[111] van Deursen JM. The role of senescent cells in ageing. Nature. 2014;509(7501):439–46. 10.1038/nature13193. 24848057 10.1038/nature13193 PMC4214092

[112] Buhl JL, Selt F, Hielscher T, Guiho R, Ecker J, Sahm F, et al. The senescence-associated secretory phenotype mediates oncogene-induced senescence in pediatric pilocytic astrocytoma. Clin Cancer Res. 2019;25(6):1851–66. 10.1158/1078-0432.CCR-18-1965. 30530705 10.1158/1078-0432.CCR-18-1965

[113] Lee S, Schmitt CA. The dynamic nature of senescence in cancer. Nat Cell Biol. 2019;21(1):94–101. 10.1038/s41556-018-0249-2. 30602768 10.1038/s41556-018-0249-2

[114] Wagner V, Gil J. An epigenetic switch: from senescent melanocytes to malignant melanoma (and Back). Cancer Cell. 2018;33(2):162–3. 10.1016/j.ccell.2018.01.013. 29438692 10.1016/j.ccell.2018.01.013

[115] Jiang WG, Sanders AJ, Katoh M, Ungefroren H, Gieseler F, Prince M, et al. Tissue invasion and metastasis: Molecular, biological and clinical perspectives. Semin Cancer Biol. 2015;35(Suppl):S244–S75. 10.1016/j.semcancer.2015.03.008. 25865774 10.1016/j.semcancer.2015.03.008

[116] Thiam A, Zhao Z, Quinn C, Barber B. Years of life lost due to metastatic melanoma in 12 countries. J Med Econ. 2016;19(3):259–64. 10.3111/13696998.2015.1115764. 26531249 10.3111/13696998.2015.1115764

[117] Beauchesne P. Extra-neural metastases of malignant gliomas: myth or reality? Cancers (Basel). 2011;3(1):461–77. 10.3390/cancers3010461. 24212625 10.3390/cancers3010461 PMC3756372

[118] Li YY, Hanna GJ, Laga AC, Haddad RI, Lorch JH, Hammerman PS. Genomic analysis of metastatic cutaneous squamous cell carcinoma. Clin Cancer Res. 2015;21(6):1447–56. 10.1158/1078-0432.CCR-14-1773. 25589618 10.1158/1078-0432.CCR-14-1773 PMC4359951

[119] Thompson AK, Kelley BF, Prokop LJ, Murad MH, Baum CL. Risk factors for cutaneous squamous cell carcinoma recurrence, metastasis, and disease-specific death: a systematic review and meta-analysis. JAMA Dermatol. 2016;152(4):419–28. 10.1001/jamadermatol.2015.4994. 26762219 10.1001/jamadermatol.2015.4994 PMC4833641

[120] Wu W, Zhong D, Zhao Z, Wang W, Li J, Zhang W. Postoperative extracranial metastasis from glioblastoma: a case report and review of the literature. World J Surg Oncol. 2017;15(1):231. 10.1186/s12957-017-1300-7. 29284526 10.1186/s12957-017-1300-7 PMC5747170

[121] Sun Q, Xu R, Xu H, Wang G, Shen X, Jiang H. Extracranial metastases of high-grade glioma: the clinical characteristics and mechanism. World J Surg Oncol. 2017;15(1):181. 10.1186/s12957-017-1249-6. 28985756 10.1186/s12957-017-1249-6 PMC5639596

[122] Undabeitia J, Castle M, Arrazola M, Pendleton C, Ruiz I, Urculo E. Multiple extraneural metastasis of glioblastoma multiforme. An Sist Sanit Navar. 2015;38(1):157–61. 10.23938/ASSN.0061. 25963474 10.23938/ASSN.0061

[123] Prasanna T, Karapetis CS, Roder D, Tie J, Padbury R, Price T, et al. The survival outcome of patients with metastatic colorectal cancer based on the site of metastases and the impact of molecular markers and site of primary cancer on metastatic pattern. Acta Oncol. 2018;57(11):1438–44. 10.1080/0284186X.2018.1487581. 30035653 10.1080/0284186X.2018.1487581

[124] Riihimaki M, Hemminki A, Fallah M, Thomsen H, Sundquist K, Sundquist J, et al. Metastatic sites and survival in lung cancer. Lung Cancer. 2014;86(1):78–84. 10.1016/j.lungcan.2014.07.020. 25130083 10.1016/j.lungcan.2014.07.020

[125] Strilic B, Offermanns S. Intravascular survival and extravasation of tumor cells. Cancer Cell. 2017;32(3):282–93. 10.1016/j.ccell.2017.07.001. 28898694 10.1016/j.ccell.2017.07.001

[126] Wu Q, Li J, Zhu S, Wu J, Chen C, Liu Q, et al. Breast cancer subtypes predict the preferential site of distant metastases: a SEER based study. Oncotarget. 2017;8(17):27990–6. 10.18632/oncotarget.15856. 28427196 10.18632/oncotarget.15856 PMC5438624

[127] Sugarbaker PH, Hoskins ER. Splenic metastases—hematogenous disease or invasive peritoneal implants? Case reports of two patients. Int J Surg Case Rep. 2020;72:266–70. 10.1016/j.ijscr.2020.05.086. 32554282 10.1016/j.ijscr.2020.05.086 PMC7303548

[128] Afrikanova I, Yeh E, Bartos D, Watowich SS, Longmore GD. Oncogene cooperativity in Friend erythroleukemia: erythropoietin receptor activation by the env gene of SFFV leads to transcriptional upregulation of PU.1, independent of SFFV proviral insertion. Oncogene. 2002;21(8):1272–84. 10.1038/sj.onc.1205183. 11850847 10.1038/sj/onc/1205183 PMC2388250

[129] Ashton JM, Balys M, Neering SJ, Hassane DC, Cowley G, Root DE, et al. Gene sets identified with oncogene cooperativity analysis regulate in vivo growth and survival of leukemia stem cells. Cell Stem Cell. 2012;11(3):359–72. 10.1016/j.stem.2012.05.024. 22863534 10.1016/j.stem.2012.05.024 PMC4023631

[130] Caswell DR, Swanton C. The role of tumour heterogeneity and clonal cooperativity in metastasis, immune evasion and clinical outcome. BMC Med. 2017;15(1):133. 10.1186/s12916-017-0900-y. 28716075 10.1186/s12916-017-0900-y PMC5514532

[131] Fang D, Chen H, Zhu JY, Wang W, Teng Y, Ding HF, et al. Epithelial-mesenchymal transition of ovarian cancer cells is sustained by Rac1 through simultaneous activation of MEK1/2 and Src signaling pathways. Oncogene. 2017;36(11):1546–58. 10.1038/onc.2016.323. 27617576 10.1038/onc.2016.323 PMC5346482

[132] Shields DJ, Murphy EA, Desgrosellier JS, Mielgo A, Lau SK, Barnes LA, et al. Oncogenic Ras/Src cooperativity in pancreatic neoplasia. Oncogene. 2011;30(18):2123–34. 10.1038/onc.2010.589. 21242978 10.1038/onc.2010.589 PMC3104672

[133] Wichmann C, Quagliano-Lo Coco I, Yildiz O, Chen-Wichmann L, Weber H, Syzonenko T, et al. Activating c-KIT mutations confer oncogenic cooperativity and rescue RUNX1/ETO-induced DNA damage and apoptosis in human primary CD34+ hematopoietic progenitors. Leukemia. 2015;29(2):279–89. 10.1038/leu.2014.179. 24897507 10.1038/leu.2014.179 PMC4320295

[134] Hobbs GA, Der CJ, Rossman KL. RAS isoforms and mutations in cancer at a glance. J Cell Sci. 2016;129(7):1287–92. 10.1242/jcs.182873. 26985062 10.1242/jcs.182873 PMC4869631

[135] Naab TJ, Gautam A, Ricks-Santi L, Esnakula AK, Kanaan YM, DeWitty RL, et al. MYC amplification in subtypes of breast cancers in African American women. BMC Cancer. 2018;18(1):274. 10.1186/s12885-018-4171-6. 29523126 10.1186/s12885-018-4171-6 PMC5845301

[136] Cao J, Li D. Searching for human oncoviruses: histories, challenges, and opportunities. Journal of cellular biochemistry. 2018;119(6):4897–906. 10.1002/jcb.26717. 29377246 10.1002/jcb.26717

[137] Chaturvedi AK, Engels EA, Pfeiffer RM, Hernandez BY, Xiao W, Kim E, et al. Human papillomavirus and rising oropharyngeal cancer incidence in the United States. J Clin Oncol. 2011;29(32):4294–301. 10.1200/JCO.2011.36.4596. 21969503 10.1200/JCO.2011.36.4596 PMC3221528

[138] Chen H, Chen XZ, Waterboer T, Castro FA, Brenner H. Viral infections and colorectal cancer: a systematic review of epidemiological studies. Int J Cancer. 2015;137(1):12–24. 10.1002/ijc.29180. 25186851 10.1002/ijc.29180

[139] Hussein HAM, Okafor IB, Walker LR, Abdel-Raouf UM, Akula SM. Cellular and viral oncogenes: the key to unlocking unknowns of Kaposi's sarcoma-associated herpesvirus pathogenesis. Arch Virol. 2018;163(10):2633–43. 10.1007/s00705-018-3918-3. 29936609 10.1007/s00705-018-3918-3

[140] Yeo-Teh NSL, Ito Y, Jha S. High-risk human papillomaviral oncogenes E6 and E7 target key cellular pathways to achieve oncogenesis. Int J Mol Sci. 2018;19(6). 10.3390/ijms19061706. 10.3390/ijms19061706 PMC6032416 29890655

[141] Yuan H, Krawczyk E, Blancato J, Albanese C, Zhou D, Wang N, et al. HPV positive neuroendocrine cervical cancer cells are dependent on Myc but not E6/E7 viral oncogenes. Sci Rep. 2017;7(1):45617. 10.1038/srep45617. 28378747 10.1038/srep45617 PMC5381214

[142] Dunford A, Weinstock DM, Savova V, Schumacher SE, Cleary JP, Yoda A, et al. Tumor-suppressor genes that escape from X-inactivation contribute to cancer sex bias. Nat Genet. 2017;49(1):10–6. 10.1038/ng.3726. 27869828 10.1038/ng.3726 PMC5206905

[143] Gao W, Li W, Xiao T, Liu XS, Kaelin WG Jr. Inactivation of the PBRM1 tumor suppressor gene amplifies the HIF-response in VHL-/- clear cell renal carcinoma. Proc Natl Acad Sci U S A. 2017;114(5):1027–32. 10.1073/pnas.1619726114. 28082722 10.1073/pnas.1619726114 PMC5293026

[144] Li L, Xu J, Qiu G, Ying J, Du Z, Xiang T, et al. Epigenomic characterization of a p53-regulated 3p22.2 tumor suppressor that inhibits STAT3 phosphorylation via protein docking and is frequently methylated in esophageal and other carcinomas. Theranostics. 2018;8(1):61–77. 10.7150/thno.20893. 29290793 10.7150/thno.20893 PMC5743460

[145] Luchini C, Veronese N, Yachida S, Cheng L, Nottegar A, Stubbs B, et al. Different prognostic roles of tumor suppressor gene BAP1 in cancer: A systematic review with meta-analysis. Genes Chromosomes Cancer. 2016;55(10):741–9. 10.1002/gcc.22381. 27223342 10.1002/gcc.22381

[146] Roa I, de Toro G, Fernandez F, Game A, Munoz S, de Aretxabala X, et al. Inactivation of tumor suppressor gene pten in early and advanced gallbladder cancer. Diagn Pathol. 2015;10(1):148. 10.1186/s13000-015-0381-2. 26294099 10.1186/s13000-015-0381-2 PMC4546176

[147] Deshmukh A, Binju M, Arfuso F, Newsholme P, Dharmarajan A. Role of epigenetic modulation in cancer stem cell fate. Int J Biochem Cell Biol. 2017;90:9–16. 10.1016/j.biocel.2017.07.003. 28711634 10.1016/j.biocel.2017.07.003

[148] Bennett RL, Licht JD. Targeting epigenetics in cancer. Annu Rev Pharmacol Toxicol. 2018;58(1):187–207. 10.1146/annurev-pharmtox-010716-105106. 28992434 10.1146/annurev-pharmtox-010716-105106 PMC5800772

[149] Flavahan WA, Gaskell E, Bernstein BE. Epigenetic plasticity and the hallmarks of cancer. Science. 2017;357(6348). 10.1126/science.aal2380. 10.1126/science.aal2380 PMC5940341 28729483

[150] Poli V, Fagnocchi L, Zippo A. Tumorigenic cell reprogramming and cancer plasticity: ınterplay between signaling, microenvironment, and epigenetics. Stem Cells Int. 2018;2018:4598195–16. 10.1155/2018/4598195. 29853913 10.1155/2018/4598195 PMC5954911

[151] Feinberg AP, Koldobskiy MA, Gondor A. Epigenetic modulators, modifiers and mediators in cancer aetiology and progression. Nat Rev Genet. 2016;17(5):284–99. 10.1038/nrg.2016.13. 26972587 10.1038/nrg.2016.13 PMC4888057

[152] Yousef MH, El-Fawal HAN, Abdelnaser A. Hepigenetics: a review of epigenetic modulators and potential therapies in hepatocellular carcinoma. Biomed Res Int. 2020;2020:9593254–30. 10.1155/2020/9593254. 33299889 10.1155/2020/9593254 PMC7707949

[153] Janssens Y, Wynendaele E, Vanden Berghe W, De Spiegeleer B. Peptides as epigenetic modulators: therapeutic implications. Clin Epigenetics. 2019;11(1):101. 10.1186/s13148-019-0700-7. 31300053 10.1186/s13148-019-0700-7 PMC6624906

[154] Singh Nanda J, Kumar R, Raghava GP. dbEM: A database of epigenetic modifiers curated from cancerous and normal genomes. Sci Rep. 2016;6(1):19340. 10.1038/srep19340. 26777304 10.1038/srep19340 PMC4726101

[155] Perduca V, Omichessan H, Baglietto L, Severi G. Mutational and epigenetic signatures in cancer tissue linked to environmental exposures and lifestyle. Curr Opin Oncol. 2018;30(1):61–7. 10.1097/CCO.0000000000000418. 29076965 10.1097/CCO.0000000000000418

[156] Wainwright EN, Scaffidi P. Epigenetics and cancer stem cells: unleashing, hijacking, and restricting cellular plasticity. Trends Cancer. 2017;3(5):372–86. 10.1016/j.trecan.2017.04.004. 28718414 10.1016/j.trecan.2017.04.004 PMC5506260

[157] Jones PA, Laird PW. Cancer epigenetics comes of age. Nat Genet. 1999;21(2):163–7. 10.1038/5947. 9988266 10.1038/5947

[158] Abbaoui B, Telu KH, Lucas CR, Thomas-Ahner JM, Schwartz SJ, Clinton SK, et al. The impact of cruciferous vegetable isothiocyanates on histone acetylation and histone phosphorylation in bladder cancer. J Proteomics. 2017;156:94–103. 10.1016/j.jprot.2017.01.013. 28132875 10.1016/j.jprot.2017.01.013 PMC5324139

[159] Lund AH, van Lohuizen M. Epigenetics and cancer. Genes Dev. 2004;18(19):2315–35. 10.1101/gad.1232504. 15466484 10.1101/gad.1232504

[160] Kang H, Wu D, Fan T, Zhu Y. Activities of chromatin remodeling factors and histone chaperones and their effects in root apical meristem development. Int J Mol Sci. 2020;21(3). 10.3390/ijms21030771. 10.3390/ijms21030771 PMC7038114 31991579

[161] Dawson MA, Kouzarides T. Cancer epigenetics: from mechanism to therapy. Cell. 2012;150(1):12–27. 10.1016/j.cell.2012.06.013. 22770212 10.1016/j.cell.2012.06.013

[162] Blackadar CB. Historical review of the causes of cancer. World J Clin Oncol. 2016;7(1):54–86. 10.5306/wjco.v7.i1.54. 26862491 10.5306/wjco.v7.i1.54 PMC4734938

[163] Focusing on the cell biology of cancer. Nat Cell Biol. 2013;15(1):1. 10.1038/ncb2667. 10.1038/ncb2667 23263367

[164] Fan X, Jin Y, Chen G, Ma X, Zhang L. Gut microbiota dysbiosis drives the development of colorectal cancer. Digestion. 2021;102(4):508–15. 10.1159/000508328. 32932258 10.1159/000508328

[165] Sheflin AM, Whitney AK, Weir TL. Cancer-promoting effects of microbial dysbiosis. Curr Oncol Rep. 2014;16(10):406. 10.1007/s11912-014-0406-0. 25123079 10.1007/s11912-014-0406-0 PMC4180221

[166] Zhao G, He F, Wu C, Li P, Li N, Deng J, et al. Betaine in Inflammation: Mechanistic Aspects and Applications. Front Immunol. 2018;9:1070. 10.3389/fimmu.2018.01070. 29881379 10.3389/fimmu.2018.01070 PMC5976740

[167] Newman AC, Maddocks ODK. One-carbon metabolism in cancer. Br J Cancer. 2017;116(12):1499–504. 10.1038/bjc.2017.118. 28472819 10.1038/bjc.2017.118 PMC5518849

[168] Rosenzweig A, Blenis J, Gomes AP. Beyond the Warburg effect: how do cancer cells regulate one-carbon metabolism? Front Cell Dev Biol. 2018;6:90. 10.3389/fcell.2018.00090. 30159313 10.3389/fcell.2018.00090 PMC6103474

[169] Barnes CE, English DM, Cowley SM. Acetylation & Co: an expanding repertoire of histone acylations regulates chromatin and transcription. Essays Biochem. 2019;63(1):97–107. 10.1042/EBC20180061. 30940741 10.1042/EBC20180061 PMC6484784

[170] Narita T, Weinert BT, Choudhary C. Functions and mechanisms of non-histone protein acetylation. Nat Rev Mol Cell Biol. 2019;20(3):156–74. 10.1038/s41580-018-0081-3. 30467427 10.1038/s41580-018-0081-3

[171] Feron O. The many metabolic sources of acetyl-CoA to support histone acetylation and influence cancer progression. Ann Transl Med. 2019;7(Suppl 8):S277. 10.21037/atm.2019.11.140. 32015996 10.21037/atm.2019.11.140 PMC6976516

[172] Zhang JJ, Fan TT, Mao YZ, Hou JL, Wang M, Zhang M, et al. Nuclear dihydroxyacetone phosphate signals nutrient sufficiency and cell cycle phase to global histone acetylation. Nat Metab. 2021;3(6):859–75. 10.1038/s42255-021-00405-8. 34140692 10.1038/s42255-021-00405-8

[173] Campit SE, Meliki A, Youngson NA, Chandrasekaran S. Nutrient sensing by histone marks: reading the metabolic histone code using tracing, omics, and modeling. Bioessays. 2020;42(9):e2000083. 10.1002/bies.202000083. 32638413 10.1002/bies.202000083 PMC11426192

[174] Bassett SA, Barnett MP. The role of dietary histone deacetylases (HDACs) inhibitors in health and disease. Nutrients. 2014;6(10):4273–301. 10.3390/nu6104273. 25322459 10.3390/nu6104273 PMC4210916

[175] Ho E, Dashwood RH. Dietary manipulation of histone structure and function. World Rev Nutr Diet. 2010;101:95–102. 10.1159/000314514. 20436256 10.1159/000314514 PMC3138488

[176] Delage B, Dashwood RH. Dietary manipulation of histone structure and function. Annu Rev Nutr. 2008;28(1):347–66. 10.1146/annurev.nutr.28.061807.155354. 18598138 10.1146/annurev.nutr.28.061807.155354 PMC2737739

[177] Ng KM, Tropini C. Visualization of gut microbiota-host ınteractions via fluorescence ın situ hybridization, lectin staining, and ımaging. J Vis Exp. 2021;173(173). 10.3791/62646. 10.3791/62646 34309601

[178] Earle KA, Billings G, Sigal M, Lichtman JS, Hansson GC, Elias JE, et al. Quantitative ımaging of gut microbiota spatial organization. Cell Host Microbe. 2015;18(4):478–88. 10.1016/j.chom.2015.09.002. 26439864 10.1016/j.chom.2015.09.002 PMC4628835

[179] Mark Welch JL, Hasegawa Y, McNulty NP, Gordon JI, Borisy GG. Spatial organization of a model 15-member human gut microbiota established in gnotobiotic mice. Proc Natl Acad Sci U S A. 2017;114(43):E9105–E14. 10.1073/pnas.1711596114. 29073107 10.1073/pnas.1711596114 PMC5664539

[180] Guz M, Jeleniewicz W, Malm A, Korona-Glowniak I. A crosstalk between diet, microbiome and microRNA in epigenetic regulation of colorectal cancer. Nutrients. 2021;13(7). 10.3390/nu13072428. 10.3390/nu13072428 PMC8308570 34371938

[181] Lee HS. The interaction between gut microbiome and nutrients on development of human disease through epigenetic mechanisms. Genomics Inform. 2019;17(3):e24. 10.5808/GI.2019.17.3.e24. 31610620 10.5808/GI.2019.17.3.e24 PMC6808642

[182] Miro-Blanch J, Yanes O. Epigenetic regulation at the ınterplay between gut microbiota and host metabolism. Front Genet. 2019;10:638. 10.3389/fgene.2019.00638. 31338107 10.3389/fgene.2019.00638 PMC6628876

[183] De Almeida CV, de Camargo MR, Russo E, Amedei A. Role of diet and gut microbiota on colorectal cancer immunomodulation. World J Gastroenterol. 2019;25(2):151–62. 10.3748/wjg.v25.i2.151. 30670906 10.3748/wjg.v25.i2.151 PMC6337022

[184] El-Sayed A, Aleya L, Kamel M. The link among microbiota, epigenetics, and disease development. Environ Sci Pollut Res Int. 2021;28(23):28926–64. 10.1007/s11356-021-13862-1. 33860421 10.1007/s11356-021-13862-1

[185] Shenderov BA. Gut indigenous microbiota and epigenetics. Microb Ecol Health Dis. 2012;23(0). 10.3402/mehd.v23i0.17195. 10.3402/mehd.v23i0.17195 PMC3744659 23990811

[186] Bultman SJ. Interplay between diet, gut microbiota, epigenetic events, and colorectal cancer. Mol Nutr Food Res. 2017;61(1). 10.1002/mnfr.201500902. 10.1002/mnfr.201500902 PMC5161716 27138454

